# Synthesis, molecular docking, and cytotoxicity of quinazolinone and dihydroquinazolinone derivatives as cytotoxic agents

**DOI:** 10.1186/s13065-022-00825-x

**Published:** 2022-05-18

**Authors:** Fahimeh Taayoshi, Aida Iraji, Ali Moazzam, Meysam Soleimani, Mehdi Asadi, Keyvan Pedrood, Mosayeb Akbari, Hafezeh Salehabadi, Bagher Larijani, Neda Adibpour, Mohammad Mahdavi

**Affiliations:** 1 Department of Medicinal Chemistry, School of Pharmacy, Zanjan University of Medical Sciences, Zanjan, Iran; 2 Stem Cells Technology Research Center, Shiraz University of Medical Sciences, Shiraz, Iran; 3 Central Research Laboratory, Shiraz University of Medical Sciences, Shiraz, Iran; 4 Endocrinology and Metabolism Research Center, Endocrinology and Metabolism Clinical Sciences Institute, Tehran University of Medical Sciences, Tehran, Iran; 5 Department of Pharmaceutical Biotechnology, School of Pharmacy, Hamadan University of Medical Science Hamadan, Hamedan, Iran; 6 Department of Medicinal Chemistry, Faculty of Pharmacy and Pharmaceutical Sciences, Research Center, Tehran University of Medical Sciences, Tehran, Iran

**Keywords:** Quinazolinone, Dihydroquinazolinone Cytotoxicity, Docking, PARPs, Synthesis

## Abstract

**Background:**

Cancer is the most cause of morbidity and mortality, and a major public health problem worldwide. In this context, two series of quinazolinone 5a–e and dihydroquinazolinone 10a–f compounds were designed, synthesized as cytotoxic agents.

**Methodology:**

All derivatives (5a–e and 10a–f) were synthesized via straightforward pathways and elucidated by FTIR, ^1^H-NMR, CHNS elemental analysis, as well as the melting point. All the compounds were evaluated for their in vitro cytotoxicity effects using the MTT assay against two human cancer cell lines (MCF-7 and HCT-116) using doxorubicin as the standard drug. The test derivatives were additionally docked into the PARP10 active site using Gold software.

**Results and discussion:**

Most of the synthesized compounds, especially 5a and 10f were found to be highly potent against both cell lines. Synthesized compounds demonstrated IC_50_ in the range of 4.87–205.9 μM against HCT-116 cell line and 14.70–98.45 μM against MCF-7 cell line compared with doxorubicin with IC_50_ values of 1.20 and 1.08 μM after 72 h, respectively, indicated the plausible activities of the synthesized compounds.

**Conclusion:**

The compounds quinazolinone 5a–e and dihydroquinazolinone 10a–f showed potential activity against cancer cell lines which can lead to rational drug designing of the cytotoxic agents.

**Supplementary Information:**

The online version contains supplementary material available at 10.1186/s13065-022-00825-x.

## Introduction

Cancer is a complex disease resulting from perturbations in multiple intracellular regulatory systems and leading to a drastic increase in the number of the cells and thus tumor formation [1–3]. The investigations reveal that cancer is the second major cause of mortality in 2015. Moreover, there were 8.7 million deaths among 17.5 million cases diagnosed with cancer globally [4]. Breast, lung, prostate, and colorectal cancers are recognized as widespread types of invasive cancer, which account for about 4 in 10 of all diagnosed cases [5]. Depending on the type and stage of cancer, the common cancer treatments are radiotherapy, hormone therapy as well as surgery, and chemotherapy. However, the central problem of the last item is the failure in the distinction between healthy and cancerous cells, which results in inevitable adverse effects on the healthy cells [6]. Along the same line, Multidrug resistance (MDR) is another major source of conflict in the treatment of cancer due to the resistance of the cancerous cells against the traditional chemotherapeutic agents [7]. Therefore, the need for finding novel ways for cancer treatment is still needed.

**Fig. 1:**
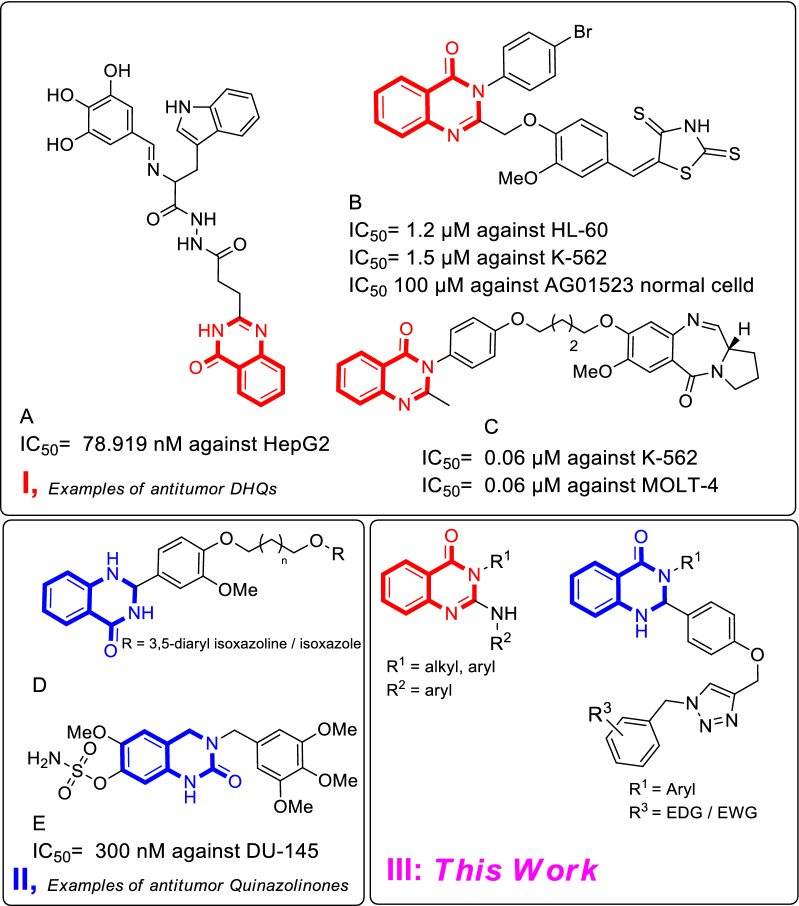
Identified representative lead candidates

Quinazoline as nitrogen-containing heterocyclic compound is synthesized in the structure of many synthetic compounds using different synthetic methods including aza-diels–alder reaction, aza-wittig reaction, metal-mediated reaction, and oxidative cyclization [8–12]. Quinazoline scaffold show diverse biologically and pharmacologically active anti-cancer [13], analgesic [14], anti-tuberculosis [15], antihypertensive [16], anti-diabetes [17] anti-melanogenesis [18, 19], anti-urease [20], antifungal [21], and antibacterial [22, 23] agents. Quinazolinone is a naturally occurring alkaloid that can be found in many natural products with diverse biological activities [24–26]. There are several quinazolinone-based compounds such as compounds A, B, and C (Fig. 1I) reported in the literature with high cytotoxicity against tested cell lines [27–29]. The inhibition of poly (ADP-ribose) polymerase 10 (PARP10) enzyme is one of the ways through which some quinazolinone analogs have demonstrated their potent anticancer activity [30, 31]. The 3,4-dihydroquinazolinone moiety is another favored scaffold due to its considerable therapeutic potential in medicinal chemistry [32, 33], mainly because of its emerging role in the treatment of cancer [34, 35]. Compounds D and E are good examples of potent antitumor activities (Fig. 1II). A bunch of methods has been proposed to synthesize 3,4-dihydroquinazolinones with plausible yields. Take the examples of the multicomponent reaction (MCR) protocols investigated by Luke R. Odell et al. [36, 37], an organo-catalyzed enantioselective approach for the synthesis of chiral trifluoromethyl dihydroquinazolinones, as a biologically important scaffold, by Xie et al. [38], and the catalyst-free and hydrophobically-directed approach for the production of functionalized 3,4-dihydroquinazolin-2(1H)-one by Chandrasekharam et al. [39].

In 2016, we disclosed a novel multi-component strategy to assemble 1,2,3-triazole derivatives of 2,3-dihydroquinazolin-4(1H)-one via click reaction with in situ prepared organic azides [40]. Furthermore, we proposed an innovative approach of Quinazolin-4(3*H*)-ones synthesis by employing CuBr and Et_3_N in 2016 [41]. With this information in hand, we focus on the synthesis of novel quinazolinone and dihydroquinazolinone to obtain more effective cytotoxic agents. All synthesized derivatives were evaluated against MCF-7 and HCT-116 cancer cell lines (Fig. 1III).

## Results and discussion

### Chemistry

**Scheme 1:**
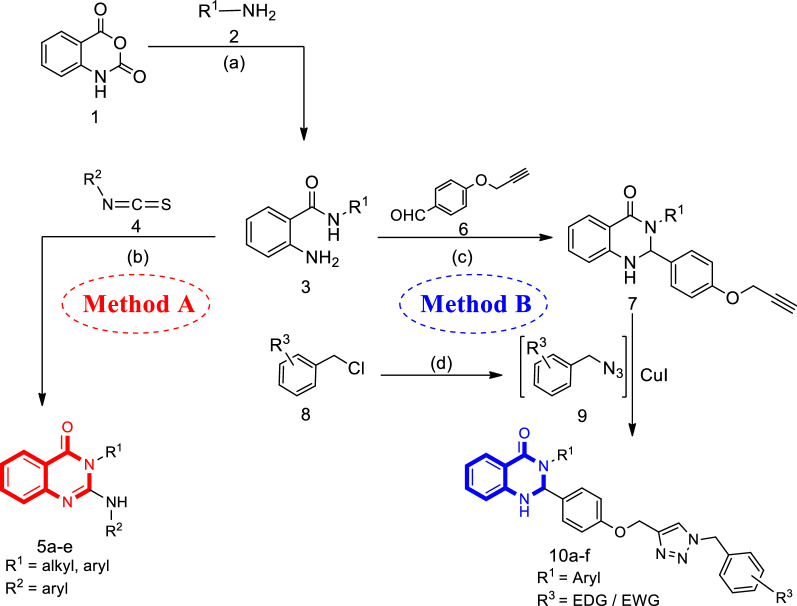
Methods for the synthesis of compounds 5a–e and 10a–f. Reagents and conditions: **a** H_2_O, r.t., 2–5 h. **b** CuBr (1 mmol), Et_3_N (1 mmol), DMF (5 ml), 80°, 8–10 h. **c** K_2_CO_3_ (1 mmol), EtOH (10 ml), reflux, 12–24 h. **d** CuI (7 mol%), H_2_O/t-BuOH (1:1), Et_3_N (1.3 mmol), r.t., 20–24 h

Two straightforward synthetic pathways were adopted to synthesize the target compounds 5a–e and 10a–f as shown in Scheme 1. The sequence for the proposed reaction initiated by treating commercially available isatoic anhydride (1) with aromatic and aliphatic amines (2) in H_2_O at room temperature to obtain the corresponding 2-aminobenzamides (3) [42]. All compounds 3 were easily prepared and used without further purifications. Next, we employed the reaction of compound 3 and phenyl isothiocyanates (4) in the presence of CuBr and Et_3_N in DMF to achieve the final product 5 (Scheme 1 Method A). The second strategy is for the synthesis of compound 10a–f in which the intermediate 7 was produced through the reaction between 2-aminobenzamides (3) and 4-(prop-2-yn-1-yloxy)benzaldehyde (6) in the presence of K_2_CO_3_ in ethanol at reflux. The presence of a triple bond in dihydroquinazolinone (7) attracted us toward click reaction to form 1,2,3-triazole ring. As a result, compound 7 was reacted with the in situ prepared (azidomethyl)benzene (9) under the Sharpless-type click reaction conditions [43]. It was found that performing the reaction in the presence of CuI (7 mol%) as the catalyst in H_2_O/t-BuOH (1:1) at room temperature within 24 h led to the formation of the corresponding product 10a–f in plausible yields (Scheme 1 Method B) according to previously reported procedures [44, 45]. The structures of final products have been verified by FT-IR, ^1^H-NMR, as well as melting point, and CHNS elemental analysis.

### Biological activity

#### Cytotoxic evaluation

The selected compounds 5a–e and 10a–f were evaluated as possible cytotoxic agents against human colon cancer HCT-116 cell line and MCF-7 breast cancer cell line by MTT assay using doxorubicin as the standard drug. As shown in Table 1, the induced cellular toxicity in the cell lines was studied at 48 and 72 h. The IC_50_ value was calculated from the inhibition rates at the mentioned durations. The analysis of variance for transformed response indicated that the cytotoxic effects of compounds depend on time, whether for the MCF-7 (Table 2) or HCT-116 (Table 3) cell lines. This is because the IC_50_ values in 72 h with p-value < 0.0001 are less than those in 48 h. Moreover, the results revealed that the IC_50_ values dramatically decreased after 72 h in comparison with 48 h of the interaction of compounds with cells.

**Table 1:** Cancer cell growth inhibitory effect of synthesized derivatives evaluated by MTT reduction assay 
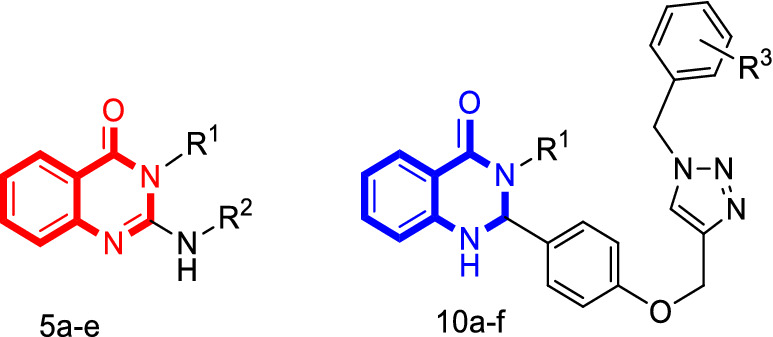

Compound	R^1^	R^2^	R^3^	IC_50_ (µM) MCF-7 48 h	IC_50_ (µM) MCF-7 72 h	IC_50_ (µM) HCT-116 48 h	IC_50_ (µM) HCT-116 72 h
5a	Ph	2-Me-C_6_H_4_	–	71.17	14.70	7.15	4.87
5b	Chloromethyl	Ph	–	101.375	76.245	59.26	37.84
5c	Cyclopropyl	Ph	–	74.92	50.40	59.24	29.15
5d	4-OMe-C_6_H_4_	Ph	–	28.84	24.99	39.22	17.76
5e	^*i*^Propyl	Ph	–	78.95	42.74	88.71	63.33
10a	Benzyl	–	H	62.29	18.88	88.79	28.99
10b	Benzyl	–	4-F	139.4	98.45	183.9	63.99
10c	Benzyl	–	4-Cl	52.00	32.30	120.35	61.02
10d	Benzyl	–	4-Br	44.68	14.80	251.1	205.9
10e	4-F-benzyl	–	2-Me	79.14	48.75	48.21	33.28
10f	4-F-benzyl	–	4-F	41.47	16.30	40.35	10.08
DOX	–	–	–	1.33	1.08	1.66	1.20

**Table 2:** Analysis of Variance for Transformed Response (λ = 0.273)

Source	DF	Adj SS	Adj MS	F-Value	p-Value
Time	1	13.005	13.0049	308.96	0.000
Compound	23	106.448	4.6282	109.95	0.000
Time*compound	23	5.271	0.2292	5.44	0.000
Error	82	3.452	0.0421		
Total	129	128.108			

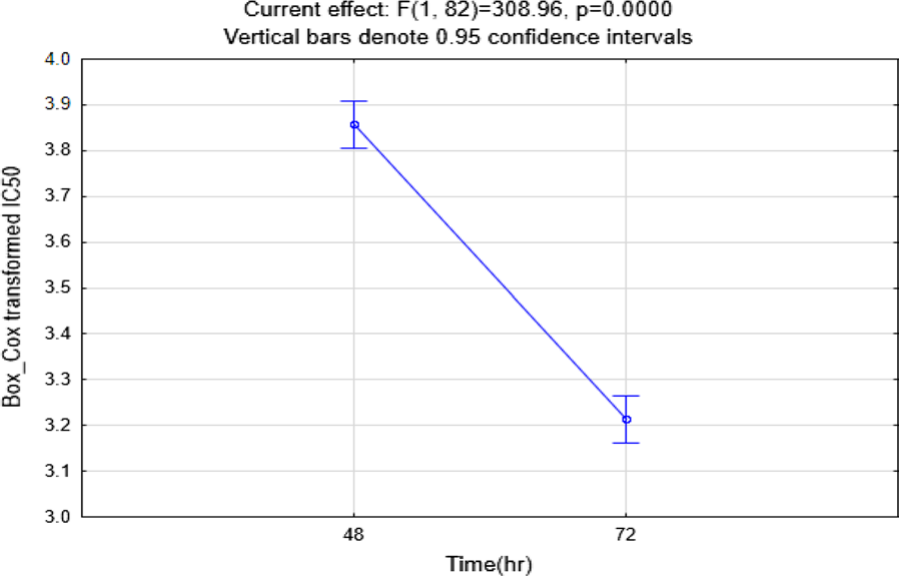

**Table 3:** Analysis of Variance for Transformed Response (λ = 0.333)

Source	DF	Adj SS	Adj MS	F-Value	p-Value
Time	1	13.832	13.8318	302.12	0.000
compound	23	143.164	6.2245	135.96	0.000
Time*compound	23	5.216	0.2268	4.95	0.000
Error	83	3.800	0.0458		
Total	130	166.298			

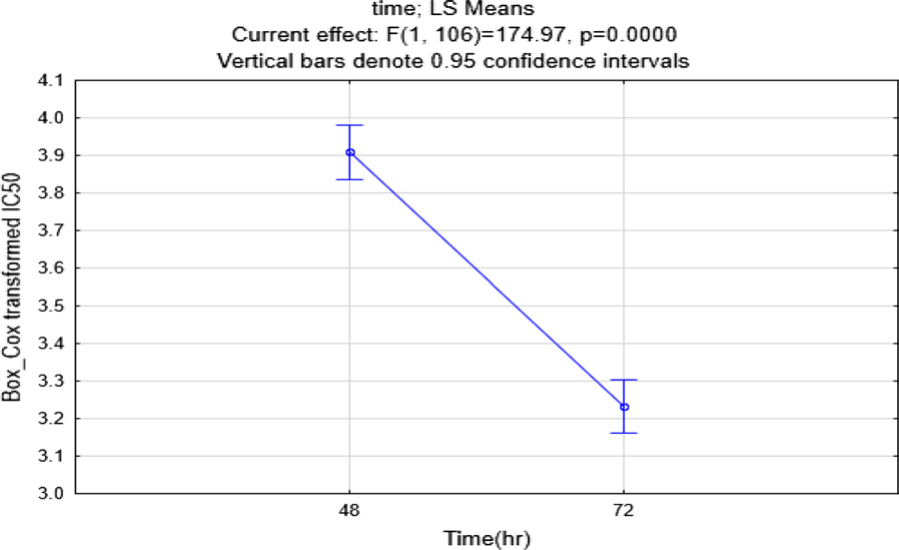

The first structure–activity relationship (SAR) explorations focused on MCF-7 cells. Assessments of 5a–e derivatives against MCF-7 demonstrated that 5d possessing R^1^ = 4-OMe-C_6_H_4_ and R^2^ = Ph afforded good potency with an IC_50_ value of 28.84 μM and 24.99 μM after 48 and 72 h followed by 5a bearing R^1^ = Ph and R^2^ = 2-Me-C_6_H_4_. It seems that increasing the bulkiness at R^1^ may improve the potency. Cytotoxic screening of 10a–f revealed that 10a as unsubstituted derivatives exhibited IC_50_ values of 62.29 μM and 18.88 μM after 48 and 72 h. The incorporation of halogen groups at R^3^ position showed different behavior so that 4-F (10b) reduced the activity compared to 10a while *para*-chlorine (10c) or *para*-bromine (10d) improved the cytotoxic potency compared to 10a. Noteworthy, the substitution of 4-F-benzyl at R^1^ position of 10b produced the most potent derivative in this set with IC_50_ values of 41.47 μM and 16.30 μM after 48 and 72 h.

With regards to the HCT-116 cancer cells, in testing the compounds 5a–e, it was shown that 5a was the most promising cytotoxic agent with IC_50_ values of 7.15 μM and 4.87 μM after 48 and 72 h. Further investigations illustrated that the replacement of Ph with other moieties at R^1^ as well as the replacement of 2-Me-C_6_H_4_ with Ph at R^2^ (5b, 5c, 5d, 5e) deteriorated the cytotoxicity potential, significantly. From the screening data of 10a-d, it was revealed that electron-withdrawing substitutions at R^3^ (10b, R^3^ = 4-F; 10c, R^3^ = 4-Cl and 10d, R^3^ = 4-Br) decrease the potency compared to 10a as unsubstituted derivative. By way of illustration 10b (R^1^ = benzyl; R^3^ = 4-F) recorded the least potency in this series with IC_50_ values of 183.9 and 63.99 μM. Interestingly, the replacement of benzyl in 10b with 4-F-benzyl moiety leads to a noticeable increase in the cytotoxicity in 10f with an IC_50_ value of 40.35 μM and 10.08 μM after 48 and 72 h.

Overall, concerning the cytotoxic evaluations on 5a–e, it can be understood that 5d was the most active derivative against MCF-7 while 5a containing Ph at R^1^ and 2-Me-C_6_H_4_ at R^2^ was the most potent cytotoxic agent against HCT-116. Assessments of 10a–f revealed that compound 10f bearing 4-F-benzyl at R^1^ and 4-F at R^3^ was the most active cytotoxic agent against both tested cell lines.

Next, to determine the safety of 5a, 5d, and 10f as the most potent derivatives on normal cell line over cancer cell lines, these derivatives were examined on Hek293 as normal cell lines by MTT reduction assay. Results were presented in Table 4. As can be seen, derivative 5a demonstrated high toxicity against Hek-293 cell lines while 5d and 10f demonstrated low toxicity in this cell line.

**Table 4:** the toxicity assessments of 5a, 5d, and 10f gainst Hek-293 cell lines

Compound	IC_50_ (µM) Hek-293 after 72 h
5a	8.71 ± 1.23
5d	68.13 ± 12.28
10f	56.11 ± 10.38
DOX	0.75 ± 0.09

#### Molecular docking

**Fig. 2:**
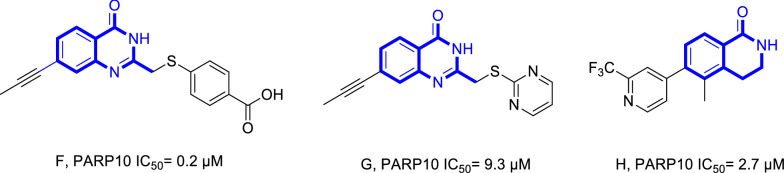
The structure of the active compound against PARP10

Poly (ADP-ribose) polymerases (PARPs) is a family of proteins involved in diverse cellular functions, especially DNA repair and maintenance of chromatin stability via ADP ribosylation. PARP10 (ARTD10) is one of the members of the PARP family that performs mono-ADP-ribosylation onto the amino acids of protein substrates from donor nicotinamide adenine dinucleotide (NAD^+^) of target proteins [46]. Recent studies have linked the activity of PARP10 to support cancer cell survival and DNA damage repairing [30]. The silencing of PARP10 in MCF7 and CaCo2 cells decreased the proliferation rate that correlated with cancer [47]. Quinazolin-4-one derivatives (Compound F, Fig. 2) were first discovered by Oregon Health and Science University as effective PARPs inhibitors involved in mono ADP-ribosylation [48, 49]. Further modification leads to the discovery of novel compounds (Compound G and H, Fig. 2) that inhibited PARP10 [50, 51]. According to the literature, the amino acids His887, Gly888, Asn910, Ala911, Tyr914, Tyr919, Ala921, Leu926, Ser927, and Tyr932 are the most important ones in the PARP10 active site [52, 53].

Regarding the similarity of reported PARP10 inhibitors with the designed structures, molecular docking evaluations were performed to study the binding mode of the most potent compounds 5a, 5d and 10f with PARP10 active site. Docking studies of the mentioned compounds were carried out using gold docking software. Validation of the molecular docking method was done by redocking the crystallographic ligand of the target enzyme, against PARP10 (PDB ID: 5LX6) which testified the validation of the docking calculations. The ChemScore fitness value of 5a, 5d, and 10f plus their interactions with residues in the PARP10 active site were documented in Table 5.

**Table 5:** Docking scores and interactions of compounds against PARP10 (PDB ID: 5LX6)

Compound	ChemScore	Interactions with key residue
5a	33.37	Ala911, Val913, Tyr914, Tyr919, Ala921, Leu926, Tyr932, Ile987
5d	28.34	His887, Ala911, Tyr919, Ala921
10f	36.96	His887, Ala911, Val913, Tyr914, Val918, Leu926, Tyr932, Ile987
Veliparib	37.89	Gly888, Tyr919, Ala921, Leu926, Ser927, Tyr932, Ile987

Alignment of the best pose of veliparib in the active site of PARP10 and crystallographic ligand recorded and RMSD value of 0.63 Å. The docked structure veliparib exhibited the interaction of this compound with Tyr919, Ala921, Leu926, Ser927, Tyr932, and Ile987 residues. Moreover, this compound showed three H-bond interactions with Gly888 and Ser927.

**Fig. 3.**
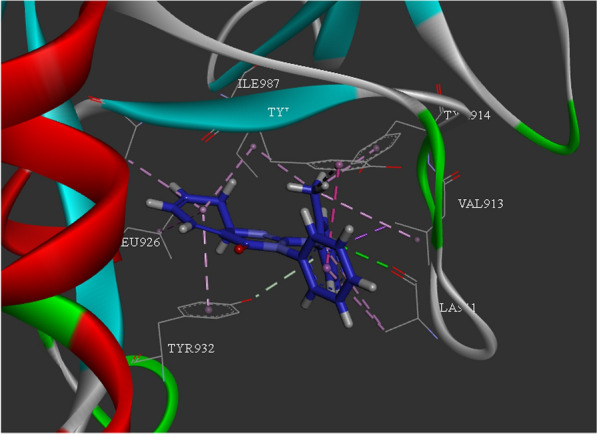
3D interaction pattern of compound 5a within PARP10 active site

Figure 3 showed the docking interactions of compound 5d within PARP10. Docking evaluation depicted four pi-alkyl interactions between the amino quinazolin-4(3H)-one ring and Ala921, Leu926, Tyr932, Ile987 as well as one hydrogen bound interaction between Ala911 and NH of amino quinazolin-4(3H)-one. 2-methylphenyl moiety exhibited one pi-sigma interaction with Val913 and one pi-alkyl interaction with Ala911 plus pi-alkyl interactions with Val913, Tyr917, Tyr919, Ile987. Also, pi-pi-T-shaped and pi-alkyl interactions were recorded between phenyl and Tyr919 and Ala911, respectively.

**Fig. 4.**
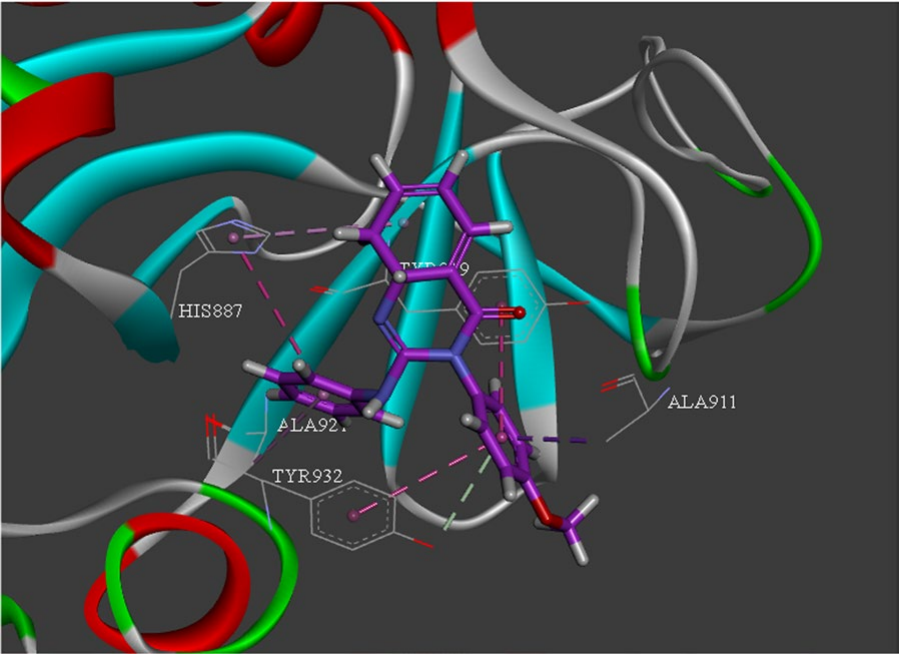
3D interaction pattern of compound 5d within PARP10 active site

According to the results of 5d docking studies (Fig. 4), the aromatic moiety of 4-methoxyphenyl presented a pi-sigma and a pi-pi-T shaped interaction with Ala911 and Tyr919, respectively. Phenyl pendant demonstrated a pi-pi-stacked interaction with His887 and a pi-alkyl interaction with Ala921. Amino-quinazolin-4(3H)-one also made a pi-alkyl interaction with Tyr919.

**Fig. 5.**
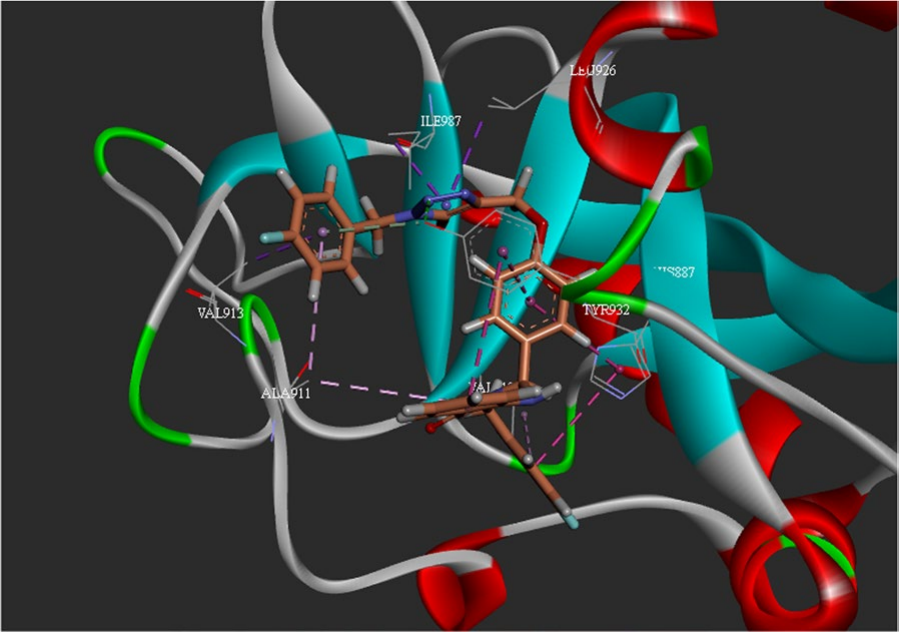
3D interaction pattern of compound 10f within PARP10 active site

The 3D interaction pattern of compound 10f (Fig. 5) showed two pi-pi-T-shaped and one pi-alkyl interactions with 4-fluorobenzyl moiety. The dihydroquinazolin-4(1H)-one ring participated in pi-pi-T-shaped and pi-alkyl interactions with Tyr932 and Ala911. Also, the phenoxy linker was fixed through pi-pi-T-shaped interaction with His887 and Typ932. Triazole ring in the middle of the molecules exhibited hydrogen bound with Typ932 plus two pi-sigma interactions with Leu926 and Ile987. Terminal 2-fluorobenzyl triazole participated in van der Waals, pi-sigma, and pi-alkyl interactions with Tyr932, Val913, Ala91, respectively.

Overall it was shown that the findings of the docking study of the most active derivatives were in line with the results of cytotoxic effects.

### Experimental

#### Materials and methods

The measured data on melting points were evaluated on a Kofler hot stage apparatus and were uncorrected. The ^1^H-NMR and IR spectra were gained by employing Bruker 400-NMR and ALPHA FT-IR spectrometer on KBr disks, respectively. The chemical reagents were obtained from Aldrich and Merck as well. Moreover, the Spectroscopic data of final products, including ^1^H-NMR and are available in the supporting information and our previous studies [41, 42].

#### Syntheses of 3-Substituted 2-(Arylamino)quinazolin-4(3*H*)-ones 5 (Method A)

The corresponding 2-aminobenzamide derivatives (3) were synthesized via the reaction of equivalent amounts of isatoic anhydride (1) and an appropriate amine (2) in water at room temperature for 2–5 h [28]. After completion of the reaction, the precipitated products were precipitated and filtered off, dried at 60 °C, and used for the further reaction without any need for more purification. Then, A mixture of 2-aminobenzamide (3) (2 mmol), isothiocyanate derivative (4) (2 mmol), CuBr (1 mmol),and Et_3_N (1 mmol) in DMF (5 ml) was heated at 80° for 8–10 h. After the reaction completion (monitored by TLC), the mixture was filtered off through a bed of Celite and washed with AcOEt. Next, H_2_O (20 ml) was added to the filtrate, it was extracted with ethyl acetate (3 × 15), and dried with Na_2_SO_4_. The solvent was then removed under reduced pressure and the crude reaction mixture was purified by column chromatography on silica gel and petroleum ether (PE)/AcOEt (5:1) as eluent. All products were recrystallized from PE/AcOEt (1:1) to give pure products 5 [44, 45].

#### General procedure for the synthesis of 3-substituted 2-[4-(prop-2-yn-1-yloxy)phenyl]-2,3-dihydroquinazolin-4(1*H*)-one derivatives 7 (Method B)

A mixture of isatoic anhydride (1) (20 mmol) and various amines (2) (20 mmol) in 50 ml water was stirred for 2–3 h at room temperature. Monitored by TLC, having completed the reactions, the resulting off-white precipitate (3) was filtered off, dried at 60 °C, and used for the next reactions without further purification [28]. Next, a mixture of 2-aminobenzamide (3) (1 mmol), 4-(prop-2-yn-1-yloxy) benzaldehyde (4) (1 mmol), and potassium carbonate (1 mmol) in 10 ml EtOH was refluxed for 12–24 h. Checked by TLC, having completed the reactions, potassium carbonate was filtered off from the reaction medium and pure product 7 was obtained as yellow crystals after the solution was cooled down to room temperature [44, 45].

#### General procedure for the synthesis of 1,2,3-triazole derivatives of 2,3-dihydroquinazolin-4(1*H*)-one 10 (Method B)

A solution of an arylmethyl chloride (8) (1.1 mmol), 0.06 gr sodium azide (0.9 mmol), and 0.13 gr Et_3_N (1.3 mmol) in 4 ml water and 4 ml *tert*-butyl alcohol was stirred at room temperature for 30 min. Next, the prepared compound 7 (0.5 mmol) and CuI (7 mol%) were added to the reaction medium, and the mixture was stirred for 20–24 h. Upon completion of the reaction, examined by TLC, the reaction mixture was diluted with 20 ml H_2_O, poured in 20 gr ice and the final product 10 was filtered of, washed with cold water, and purified by plate chromatography using silica gel and PE/EtOAc (3:1) as eluent.

#### Analytical data

2-[(2-Methylphenyl)amino]-3-phenylquinazolin-4(3*H*)-one (5a) [41]:

Yield: 77%. White crystal. M.p. 254–258 °C. IR (KBr) *υ*: 3336, 1681, 1610 cm^−1^. ^1^H NMR (400 MHz, DMSO-*d*_*6*_) *δ* 8.07 (dd, *J* = 8.0, 1.1 Hz, 1H), 7.72 (ddd, *J* = 8.2, 7.2, 1.6 Hz, 1H), 7.66–7.58 (m, 6H), 7.50 (dd, *J* = 7.5, 1.2 Hz, 1H), 7.47–7.40 (m, 3H), 7.30–7.25 (m, 1H), 7.22–7.17 (m, 1H), 2.34 (s, 3H). MS: m/z (%) = 327 [M^+^, 48%]. Anal.Calcd for C_21_H_17_N_3_O: C 77.04, H 5.23, N 12.84, Found: C 77.16, H 5.05, N 13.01.

3-(chloromethyl)-2-(phenylamino)quinazolin-4(3*H*)-one (5b):

Yield: 81%. White crystal. M.p. 194–197 °C. IR (KBr) *υ*: 3353, 1689, 1616, 780 cm^−1^. ^1^H NMR (400 MHz, CDCl_3_) *δ* 8.25 (d, *J* = 7.9 Hz, 1H), 7.64 (t, *J* = 7.8 Hz, 1H), 7.49–7.23 (m, 7H), 7.15–7.07 (m, 1H), 5.52 (s, 2H). ^13^C NMR (100 MHz, CDCl_3_) *δ* 169.71, 153.4, 144.56, 141.96, 132.19, 129.86, 129.09, 122.13, 119.75, 119.26, 118.54, 115.38, 56.57 ppm. MS: m/z (%) = 287 [M + ^2+^, 15%], 285 [M^+^, 45%]. Anal.Calcd for C_15_H_12_ClN_3_O: C 63.20, H 4.23, N 14.71, Found: C 63.16, H 3.96, N 14.92.

3-cyclopropyl-2-(phenylamino)quinazolin-4(3*H*)-one (5c):

Yield: 85%. White crystal. M.p. 141–144 °C. IR (KBr) *υ*: 3326, 1679, 1601 cm^−1^. ^1^H NMR (400 MHz, CDCl_3_) *δ* 8.16 (dd, *J* = 8.0, 1.6 Hz, 1H), 7.76 (d, *J* = 8.0 Hz, 2H), 7.63 (td, *J* = 7.7, 7.0, 1.6 Hz, 1H), 7.50–7.37 (m, 4H), 7.24 (t, *J* = 7.6 Hz, 1H), 7.18 (t, *J* = 7.4 Hz, 1H), 2.98–2.66 (m, 1H), 1.54–1.33 (m, 2H), 1.24–0.99 (m, 2H). ^13^C NMR (100 MHz, CDCl_3_) *δ* 168.81, 154.2, 144.31, 142.11, 132.08, 129.85, 129.57, 121.99, 120.03, 119.51, 118.63, 115.54, 26.30, 11.29 ppm. MS: m/z (%) = 277 [M^+^, 44%]. C_17_H_15_N_3_O: C 73.63, H 5.45, N 15.15, Found: C 73.56, H 5.76, N 14.89.

3-(4-methoxyphenyl)-2-(phenylamino)quinazolin-4(3*H*)-one (5d):

Yield: 76%. White crystal. M.p. 256–259 °C. IR (KBr) *υ*: 3348, 1675, 1608 cm^−1^. ^1^H NMR (400 MHz, DMSO-*d*_*6*_) *δ* 8.06 (dd, *J* = 8.0, 1.6 Hz, 1H), 7.76 (d, *J* = 8.0 Hz, 2H), 7.63 (td, *J* = 7.7, 7.0, 1.6 Hz, 1H), 7.50–7.37 (m, 4H), 7.27 (t, *J* = 7.6 Hz, 1H), 7.18–7.12 (m, 3H), 7.94 (d, *J* = 7.8 Hz, 2H), 3.86 (s, 3H). ^13^C NMR (100 MHz, CDCl_3_) δ 168.86, 159.6, 153.7, 145.13, 142.90, 140.11, 133.39, 130.65, 129.85, 125.10, 122.95, 121.92, 121.82, 119.86, 116.95, 114.38, 56.35 ppm. MS: m/z (%) = 343 [M^+^, 46%]. Anal.Calcd for C_21_H_17_N_3_O_2_: C 73.45, H 4.99, N 12.24, Found: C 73.32, H 5.16, N 12.39.

3-isopropyl-2-(phenylamino)quinazolin-4(3*H*)-one (5e): [41]

Yield: 80%. White crystal. M.p. 143–146 °C. IR (KBr) *υ*: 3351, 1676, 1613 cm^−1^. ^1^H NMR (400 MHz, DMSO-*d*_6_) *δ* 7.96 (d, *J* = 7.8 Hz, 1H), 7.47 (dd, *J* = 7.9, 1.6 Hz, 2H), 7.13 (ddd, *J* = 8.4, 7.1, 1.6 Hz, 1H), 6.91 (t, *J* = 7.8, 1H), 6.68 (dd, *J* = 8.2, 1.2 Hz, 1H), 6.54–6.47 (m, 2H), 6.37–6.33 (m, 2H), 4.46–3.73 (m, 1H), 1.15 (d, *J* = 6.6 Hz, 6H). MS: m/z (%) = 279 [M^+^, 47%]. Anal.Calcd for C_17_H_17_N_3_O: C 73.10, H 6.13, N 15.04, Found: C 73.39, H 5.95, N 15.24.

3-benzyl-2-(4-((1-benzyl-1*H*-1,2,3-triazol-4-yl)methoxy)phenyl)-2,3-dihydroquinazolin-4(1*H*)-one (10a): [54]

Yield: 72%. White crystal. M.p. 65–68 °C. IR (KBr) *υ*: 3390, 3058, 2929, 2840, 1655, 1610, 1230 cm^−1^. ^1^H NMR (400 MHz, DMSO-*d*_6_) *δ* 8.29 (s, 1H), 7.71 (dd, *J* = 7.7, 1.5 Hz, 1H), 7.41–7.22 (m, 14H), 7.01 (d, *J* = 8.6 Hz, 2H), 6.77–6.54 (m, 2H), 5.70 (d, *J* = 2.4 Hz, 1H), 5.62 (s, 2H), 5.31 (d, *J* = 15.3 Hz, 1H), 5.11 (s, 2H), 3.80 (d, *J* = 15.3 Hz, 1H). MS: m/z (%) = 501 [M^+^, 21%]. Anal.Calcd for C_31_H_27_N_5_O_2_: C 74.23, H 5.43, N 13.96, Found: C 74.16, H 5.25, N 13.81.

3-benzyl-2-(4-((1-(4-fluorobenzyl)-1*H*-1,2,3-triazol-4-yl)methoxy)phenyl)-2,3 dihydroquinazolin-4(1*H*)-one (10b): [54]

Yield: 77%. White crystal. M.p. 83–86 °C. IR (KBr) *υ*: 3301, 3069, 2928, 2852, 1631, 1626, 1526, 1190, 1002 cm^−1^. ^1^H NMR (400 MHz, CDCl_3_) *δ* 8.06 (d, *J* = 6.4 Hz, 1H), 7.56 (s, 1H), 7.45–7.13 (m, 14H), 6.93 (d, *J* = 8.7 Hz, 2H), 6.55 (d, *J* = 8.2 Hz, 1H), 5.61 (s, 1H), 5.57 (s, 2H), 5.28 (d, *J* = 15.3 Hz, 1H), 5.19 (s, 2H), 3.70 (d, *J* = 15.3 Hz, 1H). MS: m/z (%) = 519 [M^+^, 19%]. Anal.Calcd for C_31_H_26_FN_5_O_2_: C 71.66, H 5.04, N 13.48, Found: C 71.77, H 5.21, N 13.31.

3-benzyl-2-(4-((1-(4-chlorobenzyl)-1*H*-1,2,3-triazol-4-yl)methoxy)phenyl)-2,3-dihydroquinazolin-4(1*H*)-one (10c): [54]

Yield: 82%. White crystal. M.p. 84–87 °C. IR (KBr) *υ*: 3270, 3066, 2932, 2851, 1639, 1520, 1250, 777 cm^−1^. ^1^H NMR (400 MHz, CDCl_3_) *δ* 8.06 (dd, *J* = 7.8, 1.5 Hz, 1H), 7.56 (s, 1H), 7.50–7.36 (m, 2H), 7.37–7.25 (m, 10H), 7.26–7.17 (m, 3H), 6.93 (d, *J* = 8.7 Hz, 2H), 6.62–6.42 (m, 1H), 5.61 (d, *J* = 1.8 Hz, 1H), 5.57 (s, 2H), 5.28 (d, *J* = 15.3 Hz, 1H), 5.19 (s, 2H), 3.70 (d, *J* = 15.3 Hz, 1H). MS: m/z (%) = 537 [M + ^2+^, 6%], 535 [M^+^, 18%]. Anal.Calcd for C_31_H_26_ClN_5_O_2_: C 69.46, H 4.89, N 13.07, Found: C 69.56, H 5.05, N 13.29.

3-benzyl-2-(4-((1-(4-bromobenzyl)-1*H*-1,2,3-triazol-4-yl)methoxy)phenyl)-2,3-dihydroquinazolin-4(1*H*)-one (10d): [54]

Yield: 83%. White crystal. M.p. 93–95 °C. IR (KBr) *υ*: 3319, 3061, 2939, 2844, 1636, 1531, 1210 cm^−1^. ^1^H NMR (400 MHz, CDCl_3_) *δ* 8.06 (dd, *J* = 7.8, 1.5 Hz, 1H), 7.56 (s, 1H), 7.46–7.16 (m, 15H), 6.93 (d, *J* = 8.7 Hz, 2H), 6.54 (d, *J* = 8.0 Hz, 1H), 5.61 (d, *J* = 1.8 Hz, 1H), 5.57 (s, 2H), 5.28 (d, *J* = 15.3 Hz, 1H), 5.19 (s, 2H), 3.70 (d, *J* = 15.3 Hz, 1H). MS: m/z (%) = 581 [M + ^2+^, 15%], 579 [M^+^, 15%]. Anal.Calcd for C_31_H_26_BrN_5_O_2_: C 64.14, H 4.51, N 12.06, Found: C 64.16, H 4.45, N 11.81.

3-(4-fluorobenzyl)-2-(4-((1-(2-methylbenzyl)-1*H*-1,2,3-triazol-4-yl)methoxy)phenyl)-2,3-dihydroquinazolin-4(1*H*)-one (10e): [54]

Yield: 77%. White crystal. M.p. 63–65 °C. (KBr) *υ*: 3395, 3061, 2929, 2836, 1661, 1616, 1596, 1246, 996 cm^−1^. ^1^H NMR (400 MHz, DMSO-*d*_6_) *δ* 8.19 (s, 1H), 7.80–7.55 (m, 1H), 7.41–6.91 (m, 14H), 6.83–6.49 (m, 2H), 5.73 (d, *J* = 2.9 Hz, 1H), 5.63 (s, 2H), 5.21 (d, *J* = 15.4 Hz, 1H), 5.12 (s, 2H), 3.87 (d, *J* = 15.3 Hz, 1H), 2.32 (s, 3H). MS: m/z (%) = 533 [M^+^, 17%]. Anal.Calcd for C_32_H_28_FN_5_O_2_: C 72.03, H 5.29, N 13.12, Found: C 72.19, H 5.11, N 13.01.

3-(4-fluorobenzyl)-2-(4-((1-(4-fluorobenzyl)-1*H*-1,2,3-triazol-4-yl)methoxy)phenyl)-2,3-dihydroquinazolin-4(1*H*)-one (10f): [54]

Yield: 71%. White crystal. M.p. 69–72 °C. IR (KBr) *υ*: 3280, 3045, 2920, 2836, 1648, 1601, 1203, 1010, 965 cm^−1^. ^1^H NMR (400 MHz, DMSO-*d*_6_) *δ* 8.29 (s, 1H), 7.71 (dd, *J* = 7.8, 1.6 Hz, 1H), 7.50–7.07 (m, 12H), 7.00 (d, *J* = 8.7 Hz, 2H), 6.83–6.58 (m, 2H), 5.73 (d, *J* = 2.4 Hz, 1H), 5.61 (s, 2H), 5.20 (d, *J* = 15.3 Hz, 1H), 5.11 (s, 2H), 3.86 (d, *J* = 15.3 Hz, 1H). MS: m/z (%) = 537 [M^+^, 20%]. Anal.Calcd for C_31_H_25_F_2_N_5_O_2_: C 69.26, H 4.69, N 13.03, Found: C 69.16, H 4.46, N 12.96.

### Cytotoxic evaluation

#### Cell lines and cell culture

The human cancer cells MCF-7and HCT-116 as well as Hek-293 as normal cells were purchased from Pastor Institute of Iran. The cells were maintained in RPMI 1640 medium supplemented with 10% heat-inactivated fetal bovine serum (Company: DNAbiotec, Cat number: DB9723), and streptomycin (100 mg/mL) and penicillin (100 U/ml) at 37 °C in a humidified atmosphere with 5% CO_2_ in the air.

#### MTT assay

The cytotoxic activities of compounds 5a–e and 10a–f were evaluated against cancerous cell lines. And the most potent cytotoxic agents (5a, 5d, and 10f) against normal cell lines were examined by taking advantage of MTT (3-(4,5-dimethylthiazol-2-yl)-2,5-diphenyl tetrazolium bromide) colorimetric assay as reported method [32, 33]. The absorbance was read at 570 nm against a test wavelength of 690 nm using Graphpad Prism 8.2.1 software. The inhibition percentage of compounds was calculated as: OD_wells treated with DMSO1%_–OD_wells treated with compounds_/OD_wells treated with DMSO1%_*100 (OD = absorbance). Then, IC_50_ values were calculated by nonlinear regression analysis.

#### Molecular docking

Docking assessments of 5a, 5d, and 10f were performed using the GOLD docking program according to previously reported protocol [55, 56] The 3D-crystal structure of the PARP10 binding site (PDB ID: 5LX6) was retrieved from Protein Data Bank (http://www.rcsb.org). The protein structure was prepared using the Discovery studio client so that waters and ligands were removed from 5LX6 and all hydrogens were added. The binding site of the enzyme was defined based on the native ligand Veliparib with a 8 Å radius. For validation of docking, the ChemScore function was chosen for docking of Veliparib inside the 5LX6. All other options were set as default. After validation, 5a, 5d and 10f compounds were sketched using Hyperchem software and energy minimized by the MM1 force field. The same docking procedure was applied for docking analyses of mentioned compounds with the GOLD docking program. The top-score binding poses were used for further analysis. Protein–ligand interactions were analyzed with Discovery Studio Visualizer.

## Conclusion

In the quest for effective anticancer agents, the series of quinazolinone 5a–e and dihydroquinazolinone 10a–f were efficiently prepared and characterized. The synthetic compounds were evaluated for anticancer activity against two cell lines MCF-7 and HCT-116. Most of the compounds, especially 5a, 5d, and 10f were found to have very good activity against tested cancerous cell lines. Next safety and selectivity assessments of mentioned derivatives against normal cell lines revealed that 5d and 10f had low toxicity against Hek-293 cell lines. The molecular docking studies validated the outcome results from the anticancer activity and signified the potential of these derivatives as potent PARP10 inhibitors. As a result, these compounds can be modified further for the development of new anticancer therapeutics.

## Supplementary Information

**Additional file 1: Figure S1.** ^1^H-NMR of 2-[(2-Methylphenyl)amino]-3-phenylquinazolin-4(3*H*)-one (5a). **Figure S2.** Mass data of 2-[(2-Methylphenyl)amino]-3-phenylquinazolin-4(3*H*)-one (5a). **Figure S3.** ^1^H-NMR of 3-(chloromethyl)-2-(phenylamino)quinazolin-4(3*H*)-one (5b). **Figure S4.** Mass data of 3-(chloromethyl)-2-(phenylamino)quinazolin-4(3*H*)-one (5b). **Figure S5.** ^1^H-NMR of 3-cyclopropyl-2-(phenylamino)quinazolin-4(3*H*)-one (5c). **Figure S6.** Mass data of 3-cyclopropyl-2-(phenylamino)quinazolin-4(3*H*)-one (5c). **Figure S7.** ^1^H-NMR of 3-(4-methoxyphenyl)-2-(phenylamino)quinazolin-4(3*H*)-one (5d). **Figure S8.** Mass data of 3-(4-methoxyphenyl)-2-(phenylamino)quinazolin-4(3*H*)-one (5d). **Figure S9.** ^1^H-NMR of 3-isopropyl-2-(phenylamino)quinazolin-4(3*H*)-one (5e). **Figure S10.** Mass data of 3-isopropyl-2-(phenylamino)quinazolin-4(3*H*)-one (5e). **Figure S11.** ^1^H-NMR of 3-benzyl-2-(4-((1-benzyl-1*H*-1,2,3-triazol-4-yl)methoxy)phenyl)-2,3-dihydroquinazolin-4(1*H*)-one (10a). **Figure S12.** Mass data of 3-benzyl-2-(4-((1-benzyl-1*H*-1,2,3-triazol-4-yl)methoxy)phenyl)-2,3-dihydroquinazolin-4(1*H*)-one (10a). **Figure S13.** ^1^H-NMR of 3-benzyl-2-(4-((1-(4-fluorobenzyl)-1*H*-1,2,3-triazol-4-yl)methoxy)phenyl)-2,3 dihydroquinazolin-4(1*H*)-one (10b). **Figure S14.** Mass data of 3-benzyl-2-(4-((1-(4-fluorobenzyl)-1*H*-1,2,3-triazol-4-yl)methoxy)phenyl)-2,3 dihydroquinazolin-4(1*H*)-one (10b). **Figure S15.** ^1^H-NMR of 3-benzyl-2-(4-((1-(4-chlorobenzyl)-1*H*-1,2,3-triazol-4-yl)methoxy)phenyl)-2,3-dihydroquinazolin-4(1*H*)-one (10c). **Figure S16**. Mass data of 3-benzyl-2-(4-((1-(4-chlorobenzyl)-1*H*-1,2,3-triazol-4-yl)methoxy)phenyl)-2,3-dihydroquinazolin-4(1*H*)-one (10c). **Figure S17.** ^1^H-NMR of 3-benzyl-2-(4-((1-(4-bromobenzyl)-1*H*-1,2,3-triazol-4-yl)methoxy)phenyl)-2,3-dihydroquinazolin-4(1*H*)-one (10d). **Figure S18.** Mass data of 3-benzyl-2-(4-((1-(4-bromobenzyl)-1*H*-1,2,3-triazol-4-yl)methoxy)phenyl)-2,3-dihydroquinazolin-4(1*H*)-one (10d). **Figure S19.** ^1^H-NMR of 3-(4-fluorobenzyl)-2-(4-((1-(2-methylbenzyl)-1*H*-1,2,3-triazol-4-yl)methoxy)phenyl)-2,3-dihydroquinazolin-4(1*H*)-one (10e). **Figure S20.** Mass of 3-(4-fluorobenzyl)-2-(4-((1-(2-methylbenzyl)-1*H*-1,2,3-triazol-4-yl)methoxy)phenyl)-2,3-dihydroquinazolin-4(1*H*)-one (10e). **Figure S21.** ^1^H-NMR of 3-(4-fluorobenzyl)-2-(4-((1-(4-fluorobenzyl)-1*H*-1,2,3-triazol-4-yl)methoxy)phenyl)-2,3-dihydroquinazolin-4(1*H*)-one (10f). **Figure S21.** Mass of 3-(4-fluorobenzyl)-2-(4-((1-(4-fluorobenzyl)-1*H*-1,2,3-triazol-4-yl)methoxy)phenyl)-2,3-dihydroquinazolin-4(1*H*)-one (10f).

## Data Availability

The datasets generated and/or analysed during the current study are available in the Worldwide Protein Data Bank (wwPDB) repository. (http://www.rcsb.org).

## Abbreviations

MDR: Multidrug resistance
MCR: Multicomponent reaction
MTT: 3-(4,5-Dimethylthiazol-2-yl)-2,5-diphenyl tetrazolium bromide
OD: Optical Density
2D: 2-Dimensional
3D: 3-Dimensional
RPMI 1640: Roswell Park Memorial Institute1640
Et_3_N: Triethylamine
DMF: Dimethylformamide
TLC: Thin-layer chromatography
IC_50_: Half-maximal inhibitory concentration
