# Supplementary material for: Synthesis, molecular docking, and cytotoxicity of quinazolinone and dihydroquinazolinone derivatives as cytotoxic agents
Source: BMC Chem. 2022 May 18;16(1):35. doi: 10.1186/s13065-022-00825-x (PMC9118628; doi:10.1186/s13065-022-00825-x)
Supplement: Supplementary file 1 — Additional file 1: Figure S1. 1H-NMR of 2-[(2-Methylphenyl)amino]-3-phenylquinazolin-4(3H)-one (5a). Figure S2. Mass data of 2-[(2-Methylphenyl)amino]-3-phenylquinazolin-4(3H)-one (5a). Figure S3. 1H-NMR of 3-(chloromethyl)-2-(phenylamino)quinazolin-4(3H)-one (5b). Figure S4. Mass data of 3-(chloromethyl)-2-(phenylamino)quinazolin-4(3H)-one (5b). Figure S5. 1H-NMR of 3-cyclopropyl-2-(phenylamino)quinazolin-4(3H)-one (5c). Figure S6. Mass data of 3-cyclopropyl-2-(phenylamino)quinazolin-4(3H)-one (5c). Figure S7. 1H-NMR of 3-(4-methoxyphenyl)-2-(phenylamino)quinazolin-4(3H)-one (5d). Figure S8. Mass data of 3-(4-methoxyphenyl)-2-(phenylamino)quinazolin-4(3H)-one (5d). Figure S9. 1H-NMR of 3-isopropyl-2-(phenylamino)quinazolin-4(3H)-one (5e). Figure S10. Mass data of 3-isopropyl-2-(phenylamino)quinazolin-4(3H)-one (5e). Figure S11. 1H-NMR of 3-benzyl-2-(4-((1-benzyl-1H-1,2,3-triazol-4-yl)methoxy)phenyl)-2,3-dihydroquinazolin-4(1H)-one (10a). Figure S12. Mass data of 3-benzyl-2-(4-((1-benzyl-1H-1,2,3-triazol-4-yl)methoxy)phenyl)-2,3-dihydroquinazolin-4(1H)-one (10a). Figure S13. 1H-NMR of 3-benzyl-2-(4-((1-(4-fluorobenzyl)-1H-1,2,3-triazol-4-yl)methoxy)phenyl)-2,3 dihydroquinazolin-4(1H)-one (10b). Figure S14. Mass data of 3-benzyl-2-(4-((1-(4-fluorobenzyl)-1H-1,2,3-triazol-4-yl)methoxy)phenyl)-2,3 dihydroquinazolin-4(1H)-one (10b). Figure S15. 1H-NMR of 3-benzyl-2-(4-((1-(4-chlorobenzyl)-1H-1,2,3-triazol-4-yl)methoxy)phenyl)-2,3-dihydroquinazolin-4(1H)-one (10c). Figure S16. Mass data of 3-benzyl-2-(4-((1-(4-chlorobenzyl)-1H-1,2,3-triazol-4-yl)methoxy)phenyl)-2,3-dihydroquinazolin-4(1H)-one (10c). Figure S17. 1H-NMR of 3-benzyl-2-(4-((1-(4-bromobenzyl)-1H-1,2,3-triazol-4-yl)methoxy)phenyl)-2,3-dihydroquinazolin-4(1H)-one (10d). Figure S18. Mass data of 3-benzyl-2-(4-((1-(4-bromobenzyl)-1H-1,2,3-triazol-4-yl)methoxy)phenyl)-2,3-dihydroquinazolin-4(1H)-one (10d). Figure S19. 1H-NMR of 3-(4-fluorobenzyl)-2-(4-((1-(2-methylbenzyl)-1H-1,2,3-triazol-4-yl)methoxy)phenyl)-2,3- [file 13065_2022_825_MOESM1_ESM.docx]

**Figure legends**

**Fig. S1.** ^1^H-NMR of 2-[(2-Methylphenyl)amino]-3-phenylquinazolin-4(3*H*)-one (5a)

**Fig. S2.** Mass data of 2-[(2-Methylphenyl)amino]-3-phenylquinazolin-4(3*H*)-one (5a)

**Fig. S3.** ^1^H-NMR of 3-(chloromethyl)-2-(phenylamino)quinazolin-4(3*H*)-one (5b)

**Fig. S4.** Mass data of 3-(chloromethyl)-2-(phenylamino)quinazolin-4(3*H*)-one (5b)

**Fig. S5.** ^1^H-NMR of 3-cyclopropyl-2-(phenylamino)quinazolin-4(3*H*)-one (5c)

**Fig. S6.** Mass data of 3-cyclopropyl-2-(phenylamino)quinazolin-4(3*H*)-one (5c)

**Fig. S7.** ^1^H-NMR of 3-(4-methoxyphenyl)-2-(phenylamino)quinazolin-4(3*H*)-one (5d)

**Fig. S8.** Mass data of 3-(4-methoxyphenyl)-2-(phenylamino)quinazolin-4(3*H*)-one (5d)

**Fig. S9.** ^1^H-NMR of 3-isopropyl-2-(phenylamino)quinazolin-4(3*H*)-one (5e)

**Fig. S10.** Mass data of 3-isopropyl-2-(phenylamino)quinazolin-4(3*H*)-one (5e)

**Fig. S11.** ^1^H-NMR of 3-benzyl-2-(4-((1-benzyl-1*H*-1,2,3-triazol-4-yl)methoxy)phenyl)-2,3-dihydroquinazolin-4(1*H*)-one (10a)

**Fig. S12.** Mass data of 3-benzyl-2-(4-((1-benzyl-1*H*-1,2,3-triazol-4-yl)methoxy)phenyl)-2,3-dihydroquinazolin-4(1*H*)-one (10a)

**Fig. S13.** ^1^H-NMR of 3-benzyl-2-(4-((1-(4-fluorobenzyl)-1*H*-1,2,3-triazol-4-yl)methoxy)phenyl)-2,3 dihydroquinazolin-4(1*H*)-one (10b)

**Fig. S14.** Mass data of 3-benzyl-2-(4-((1-(4-fluorobenzyl)-1*H*-1,2,3-triazol-4-yl)methoxy)phenyl)-2,3 dihydroquinazolin-4(1*H*)-one (10b)

**Fig. S15.** ^1^H-NMR of 3-benzyl-2-(4-((1-(4-chlorobenzyl)-1*H*-1,2,3-triazol-4-yl)methoxy)phenyl)-2,3-dihydroquinazolin-4(1*H*)-one (10c)

**Fig. S16.** Mass data of 3-benzyl-2-(4-((1-(4-chlorobenzyl)-1*H*-1,2,3-triazol-4-yl)methoxy)phenyl)-2,3-dihydroquinazolin-4(1*H*)-one (10c)

**Fig. S17.** ^1^H-NMR of 3-benzyl-2-(4-((1-(4-bromobenzyl)-1*H*-1,2,3-triazol-4-yl)methoxy)phenyl)-2,3-dihydroquinazolin-4(1*H*)-one (10d)

**Fig. S18.** Mass data of 3-benzyl-2-(4-((1-(4-bromobenzyl)-1*H*-1,2,3-triazol-4-yl)methoxy)phenyl)-2,3-dihydroquinazolin-4(1*H*)-one (10d)

**Fig. S19.** ^1^H-NMR of 3-(4-fluorobenzyl)-2-(4-((1-(2-methylbenzyl)-1*H*-1,2,3-triazol-4-yl)methoxy)phenyl)-2,3-dihydroquinazolin-4(1*H*)-one (10e)

**Fig. S20.** Mass of 3-(4-fluorobenzyl)-2-(4-((1-(2-methylbenzyl)-1*H*-1,2,3-triazol-4-yl)methoxy)phenyl)-2,3-dihydroquinazolin-4(1*H*)-one (10e)

**Fig. S21.** ^1^H-NMR of 3-(4-fluorobenzyl)-2-(4-((1-(4-fluorobenzyl)-1*H*-1,2,3-triazol-4-yl)methoxy)phenyl)-2,3-dihydroquinazolin-4(1*H*)-one (10f)

**Fig. S21.** Mass of 3-(4-fluorobenzyl)-2-(4-((1-(4-fluorobenzyl)-1*H*-1,2,3-triazol-4-yl)methoxy)phenyl)-2,3-dihydroquinazolin-4(1*H*)-one (10f)


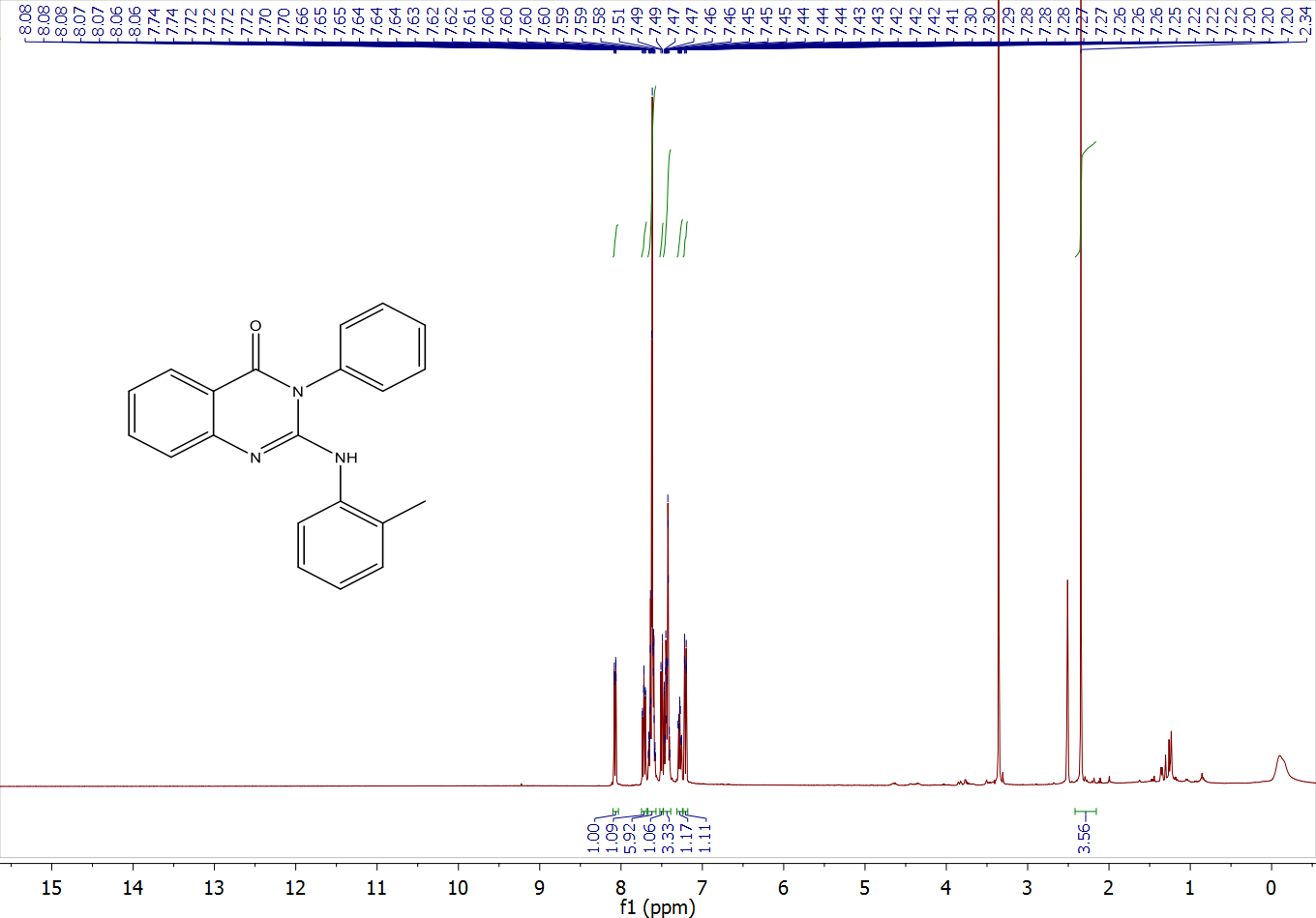


**Fig. S1.** ^1^H-NMR of 2-[(2-Methylphenyl)amino]-3-phenylquinazolin-4(3*H*)-one (5a)


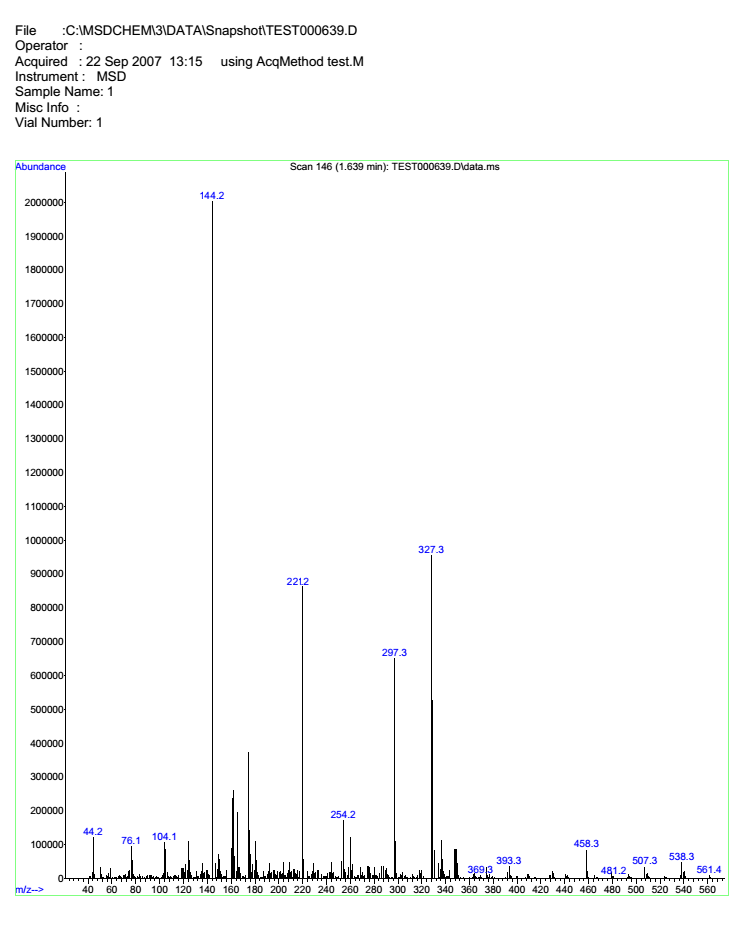


**Fig. S2.** Mass data of 2-[(2-Methylphenyl)amino]-3-phenylquinazolin-4(3*H*)-one (5a)


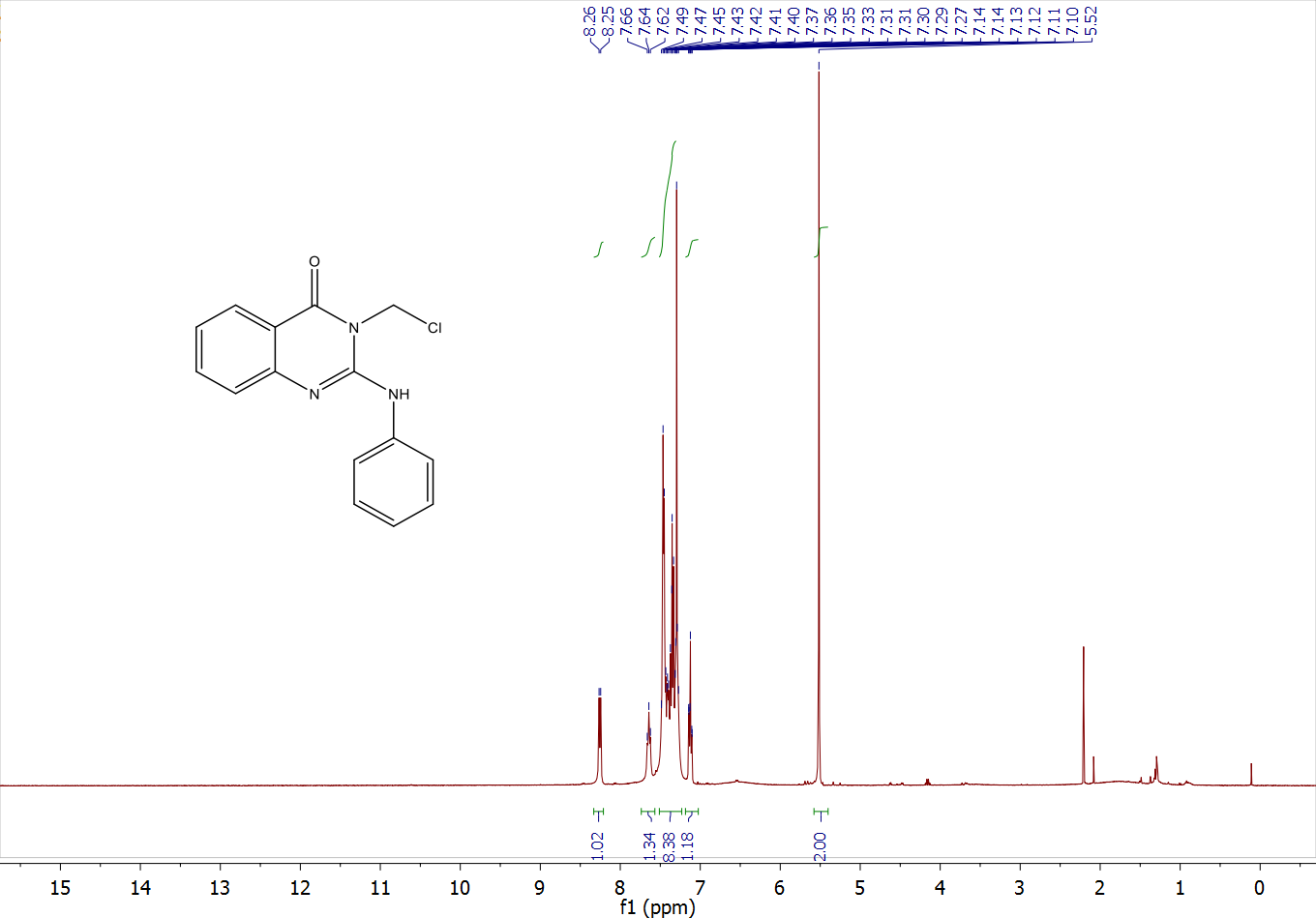


**Fig. S3.** ^1^H-NMR of 3-(chloromethyl)-2-(phenylamino)quinazolin-4(3*H*)-one (5b)

^
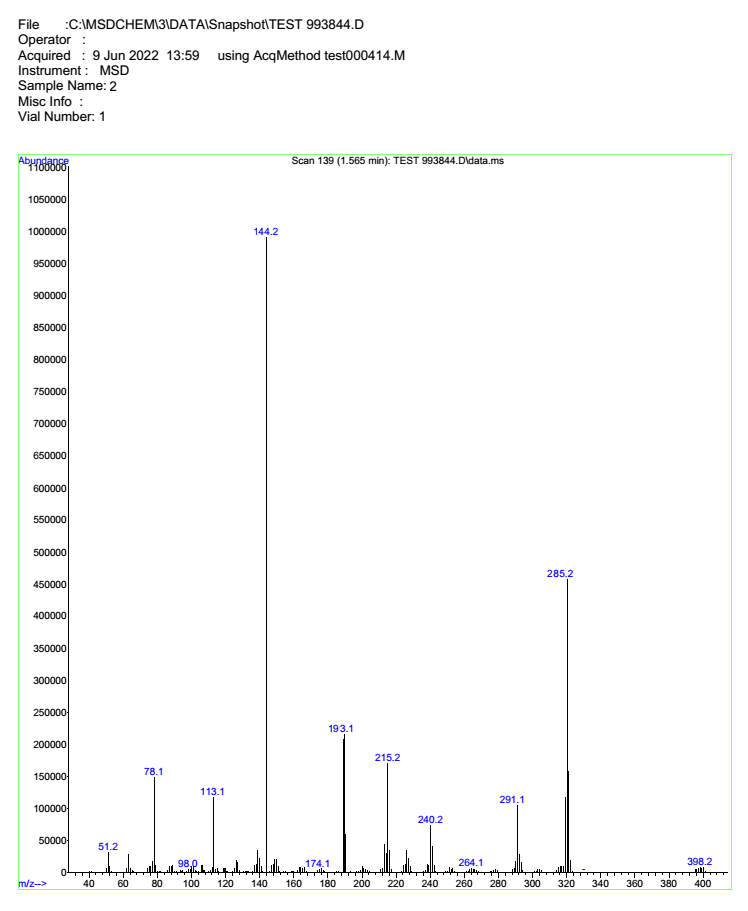
^

**Fig. S4.** Mass of 3-(chloromethyl)-2-(phenylamino)quinazolin-4(3*H*)-one (5b)

^
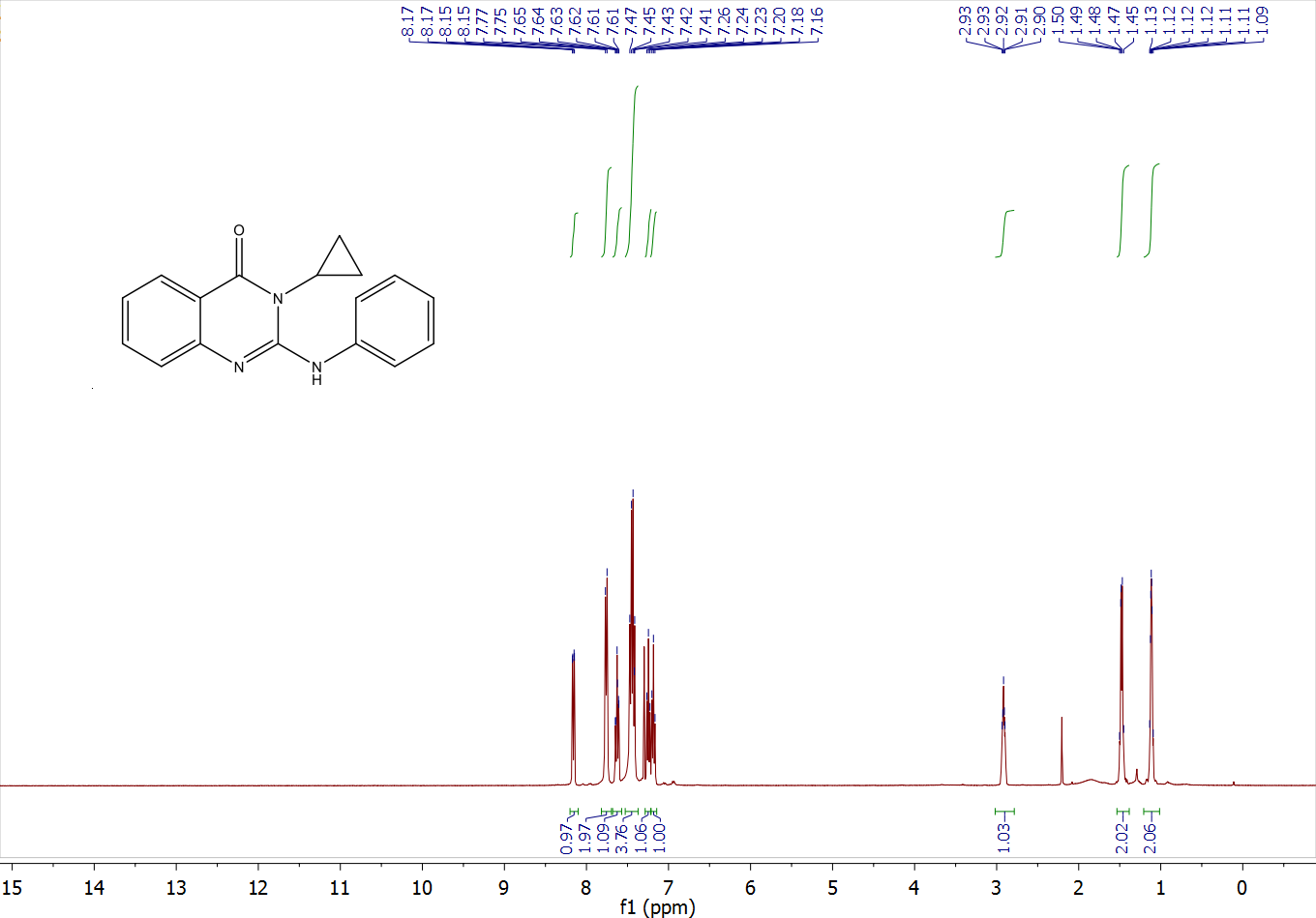
^

**Fig. S5.** ^1^H-NMR of 3-cyclopropyl-2-(phenylamino)quinazolin-4(3*H*)-one (5c)

^
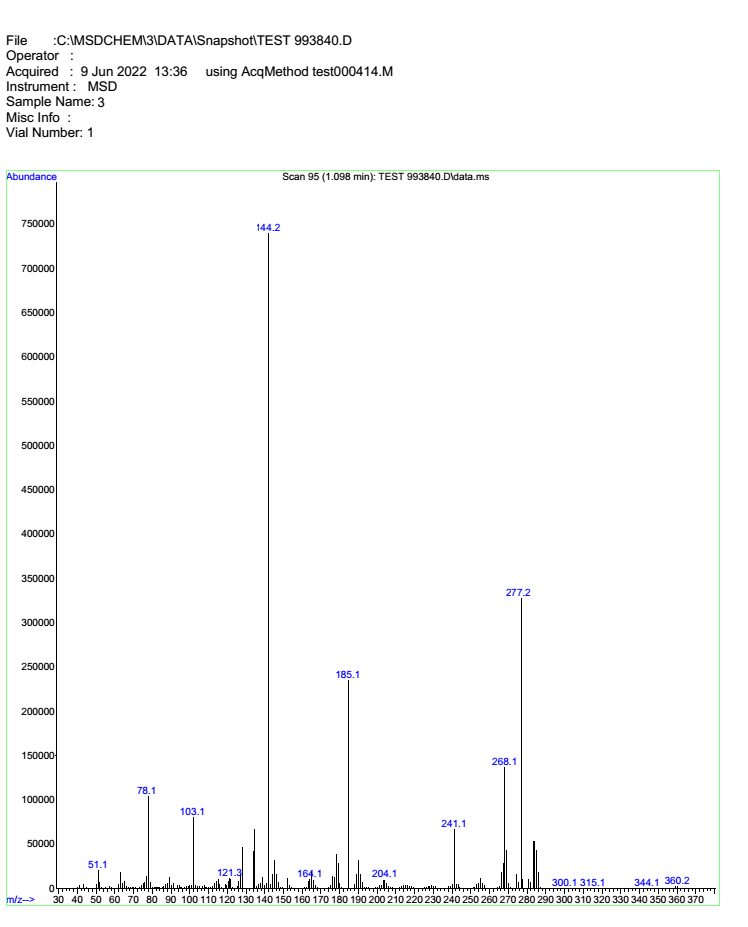
^

**Fig. S6.** Mass of 3-cyclopropyl-2-(phenylamino)quinazolin-4(3*H*)-one (5c)

^
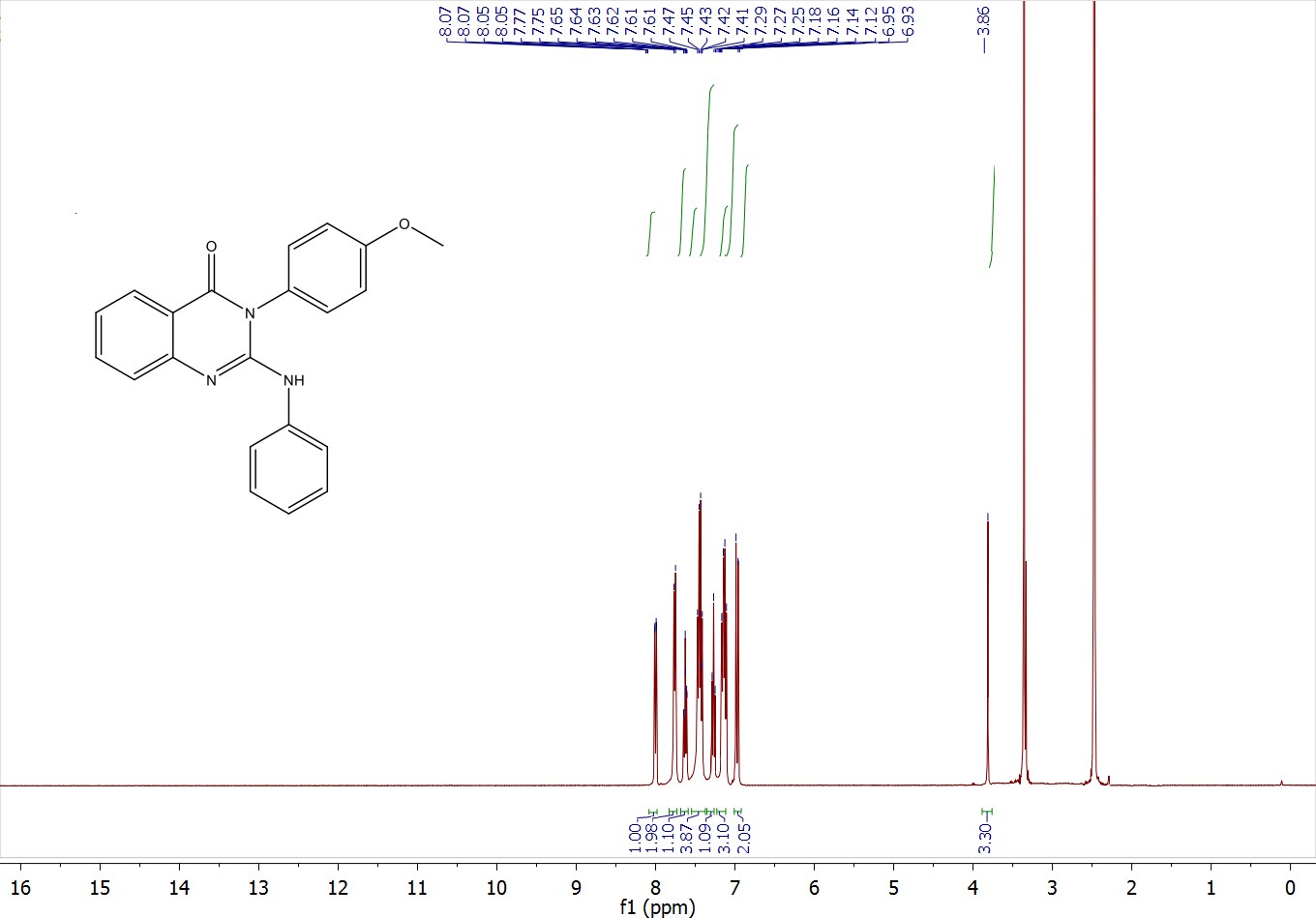
^

**Fig. S7.** ^1^H-NMR of 3-(4-methoxyphenyl)-2-(phenylamino)quinazolin-4(3*H*)-one (5d)

^
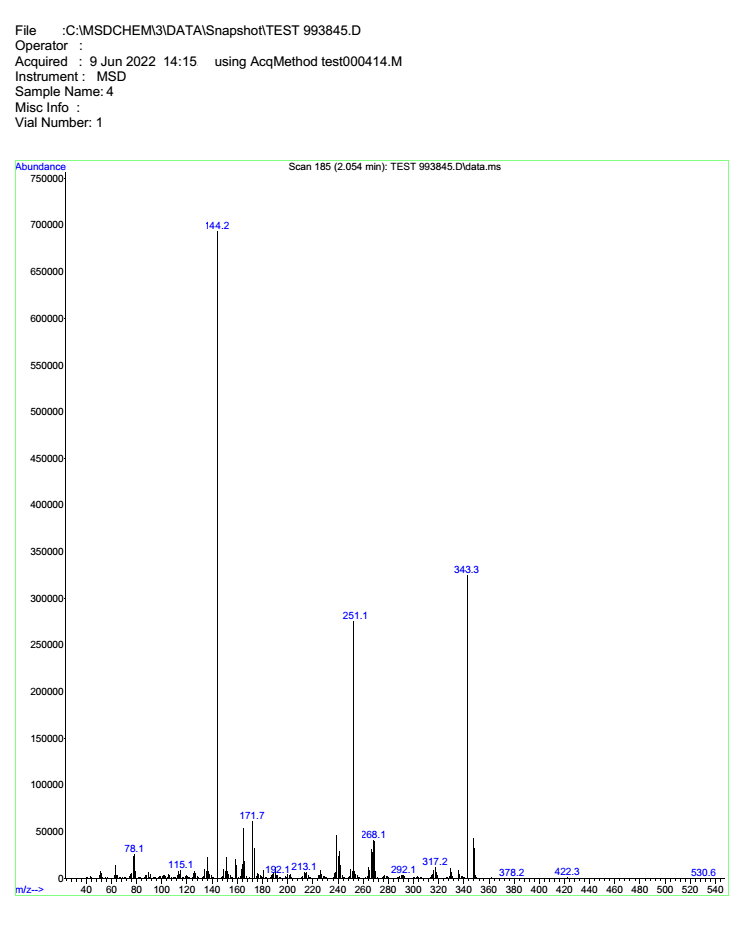
^

**Fig. S8.** Mass of 3-(4-methoxyphenyl)-2-(phenylamino)quinazolin-4(3*H*)-one (5d)

^
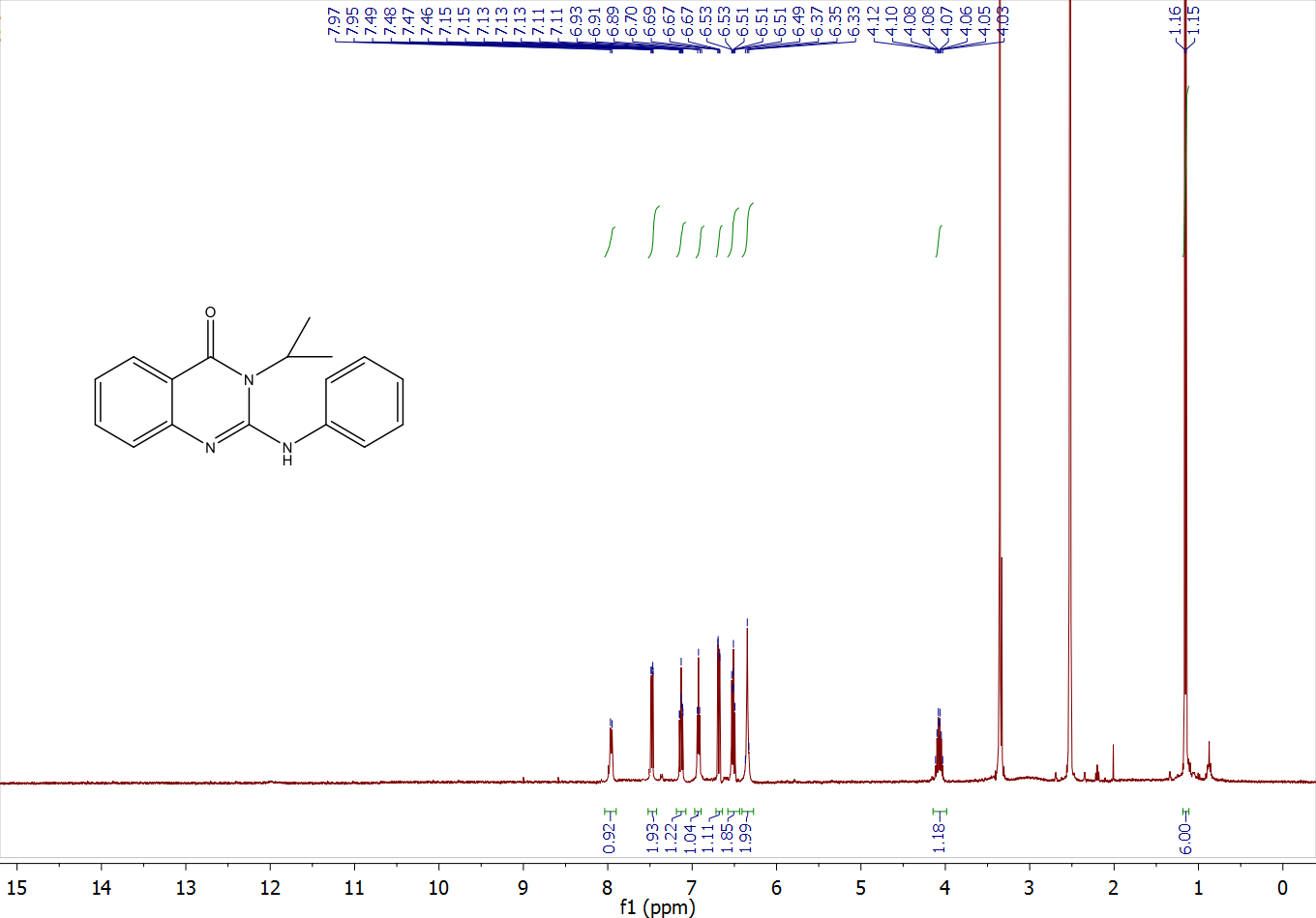
^

**Fig. S9.** ^1^H-NMR of 3-isopropyl-2-(phenylamino)quinazolin-4(3*H*)-one (5e)

^
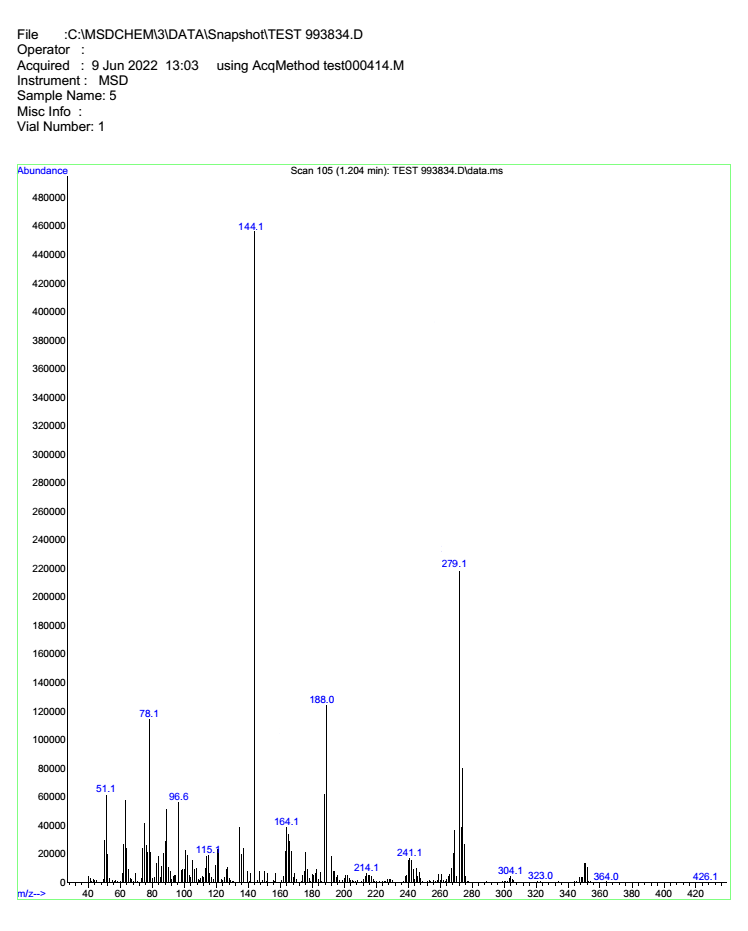
^

**Fig. S10.** Mass of 3-isopropyl-2-(phenylamino)quinazolin-4(3*H*)-one (5e)


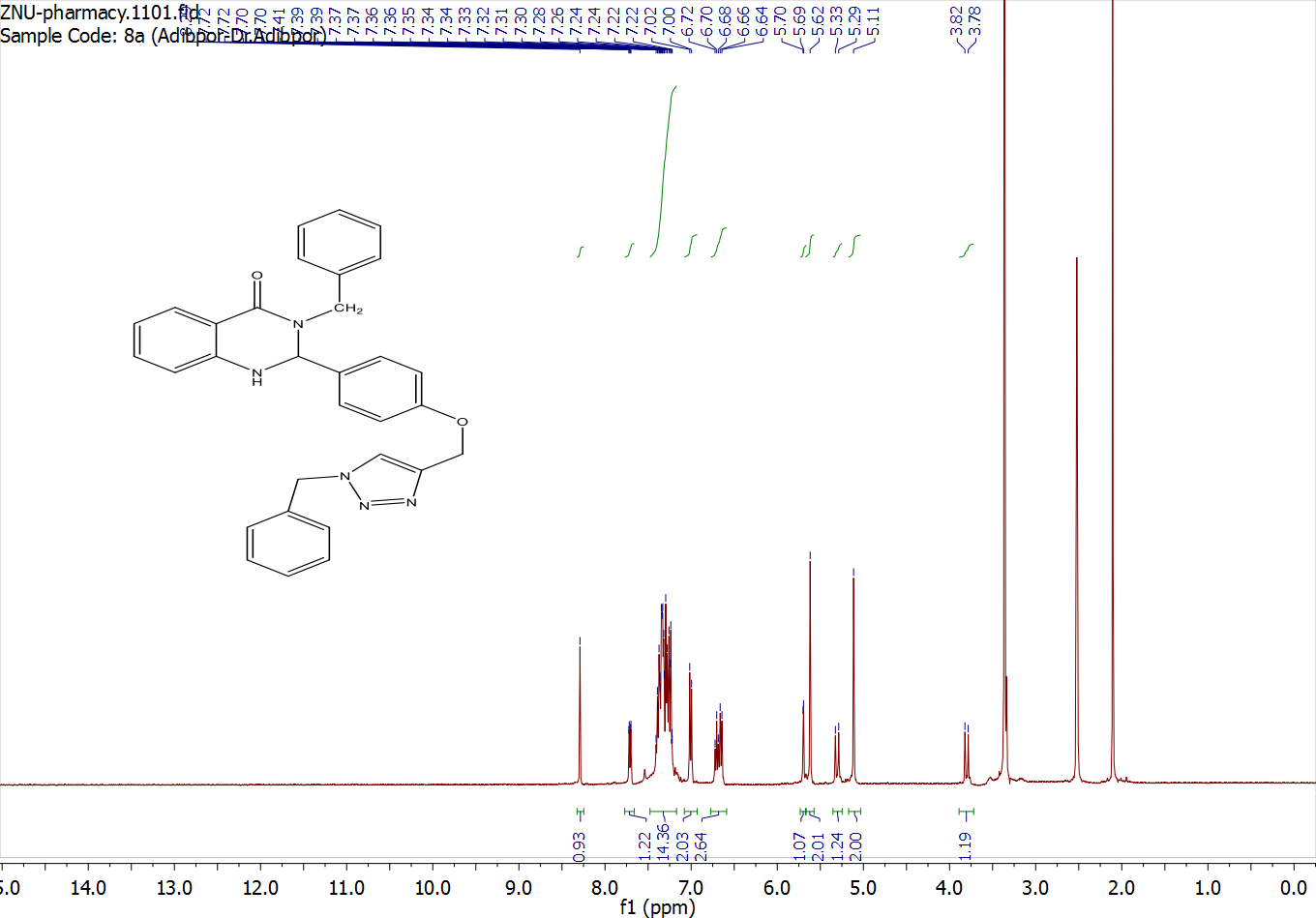


**Fig. S11.** ^1^H-NMR of 3-benzyl-2-(4-((1-benzyl-1*H*-1,2,3-triazol-4-yl)methoxy)phenyl)-2,3-dihydroquinazolin-4(1*H*)-one (10a)


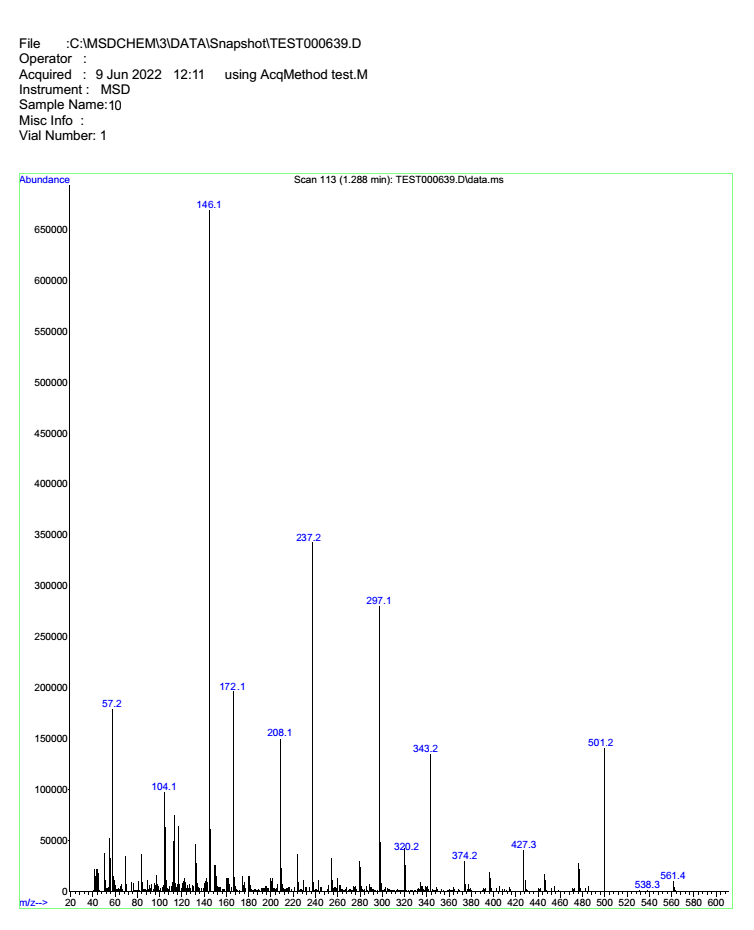


**Fig. S12.** Mass of 3-benzyl-2-(4-((1-benzyl-1*H*-1,2,3-triazol-4-yl)methoxy)phenyl)-2,3-dihydroquinazolin-4(1*H*)-one (10a)


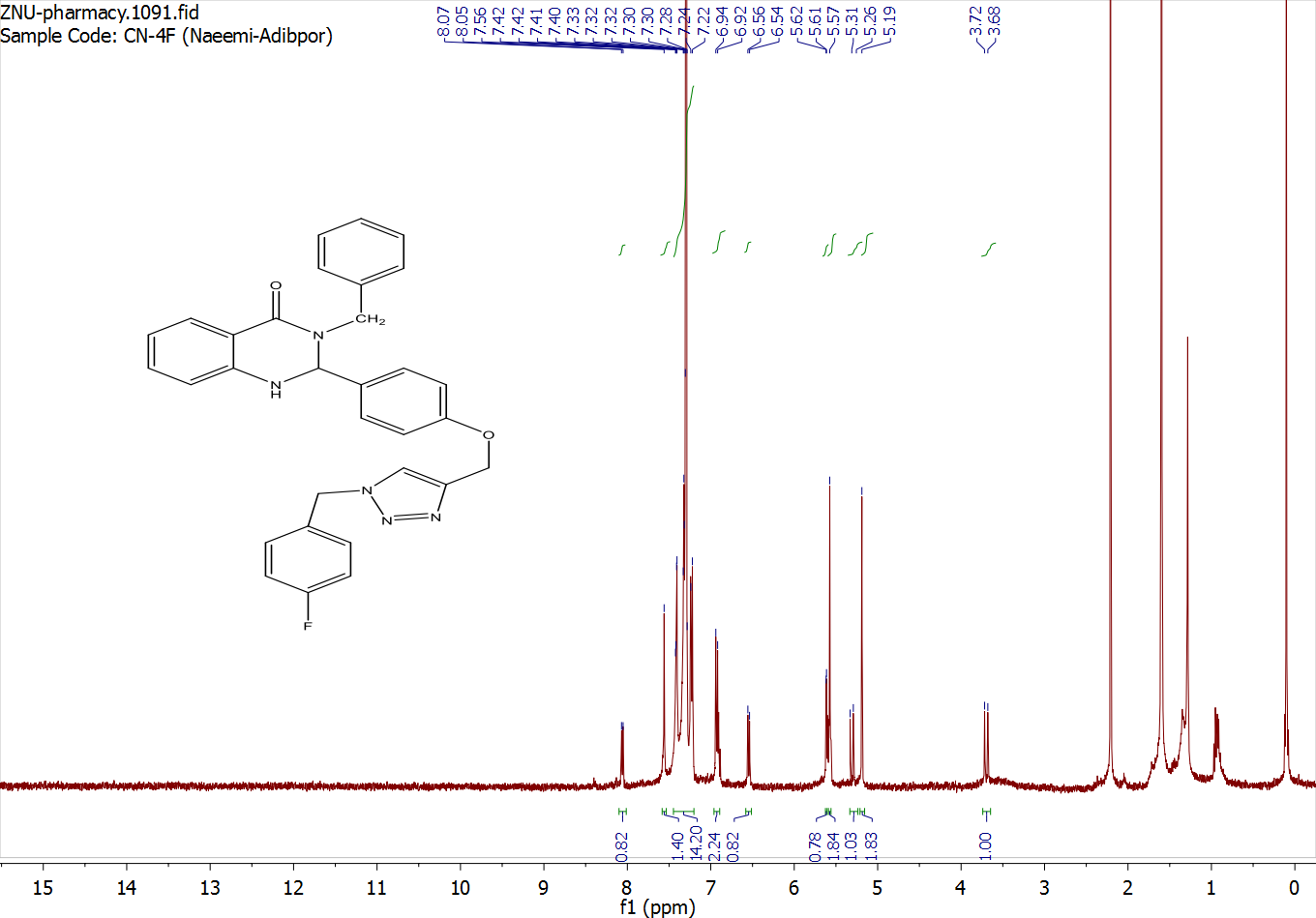


**Fig. S13.** ^1^H-NMR of 3-benzyl-2-(4-((1-(4-fluorobenzyl)-1*H*-1,2,3-triazol-4-yl)methoxy)phenyl)-2,3 dihydroquinazolin-4(1*H*)-one (10b)


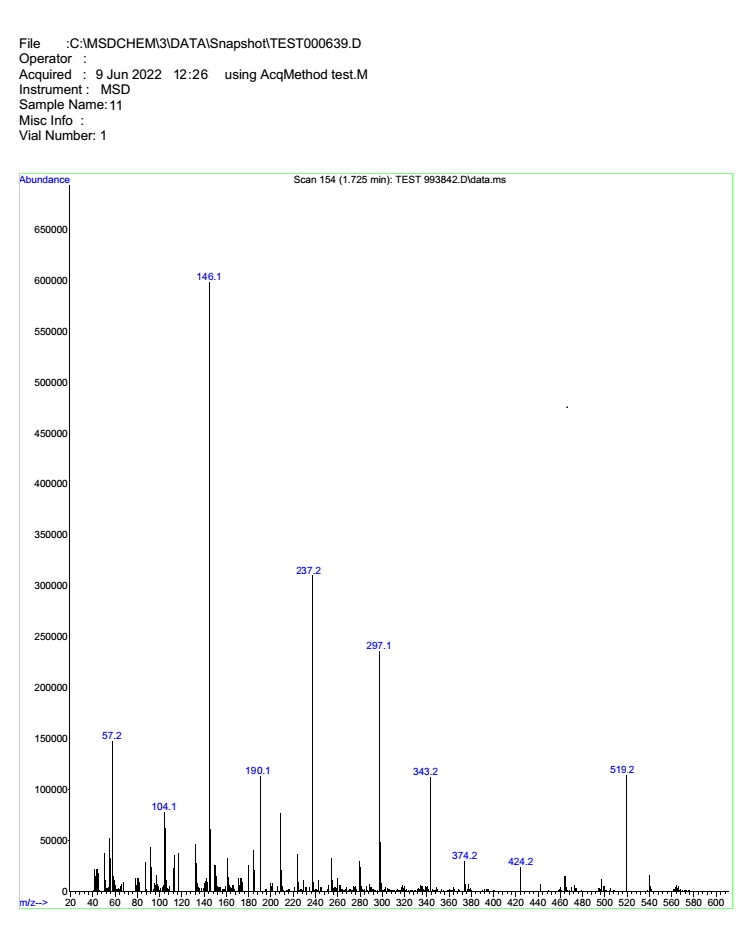


**Fig. S14.** Mass of 3-benzyl-2-(4-((1-(4-fluorobenzyl)-1*H*-1,2,3-triazol-4-yl)methoxy)phenyl)-2,3 dihydroquinazolin-4(1*H*)-one (10b)


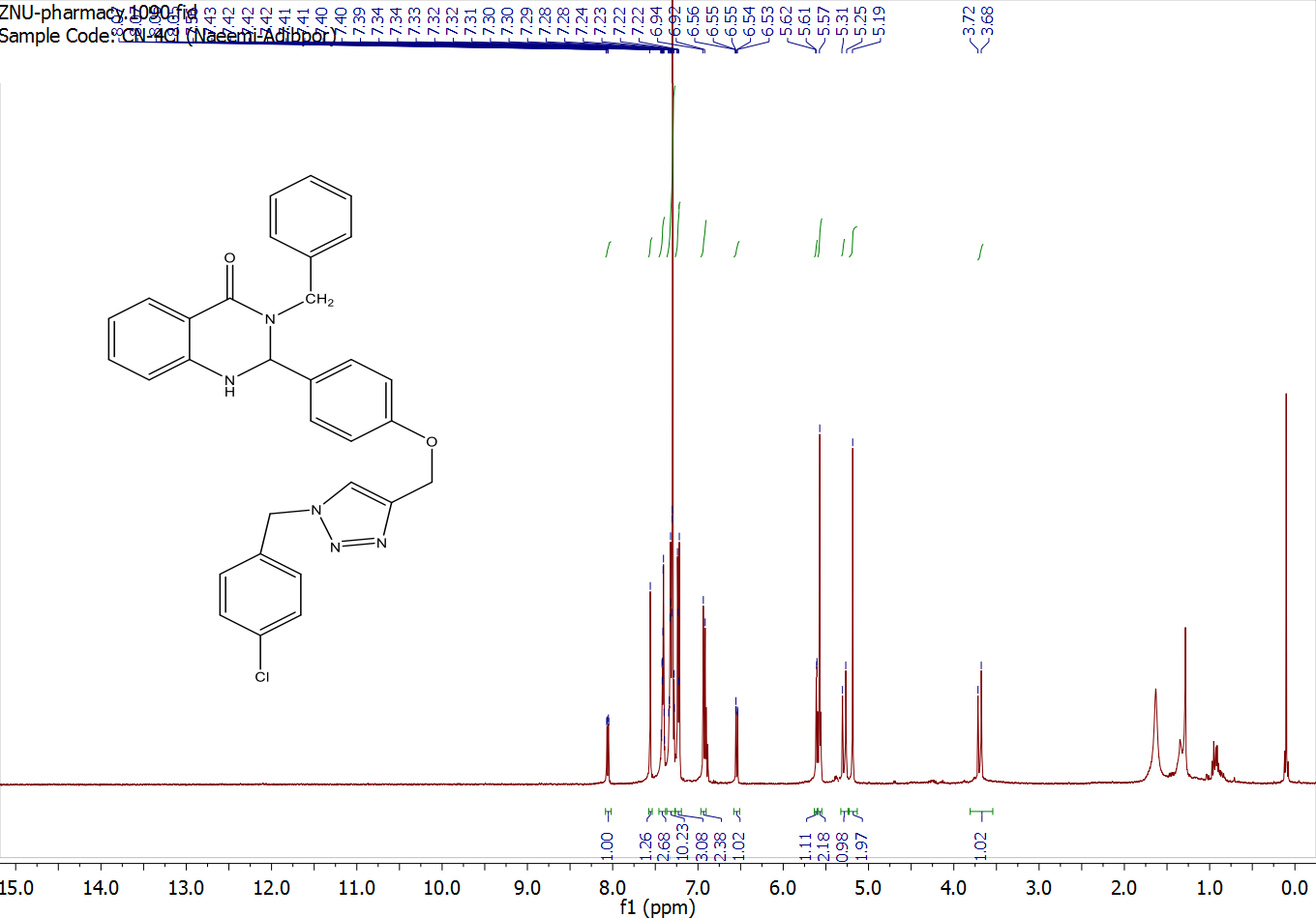


**Fig. S15.** ^1^H-NMR of 3-benzyl-2-(4-((1-(4-chlorobenzyl)-1*H*-1,2,3-triazol-4-yl)methoxy)phenyl)-2,3-dihydroquinazolin-4(1*H*)-one (10c)


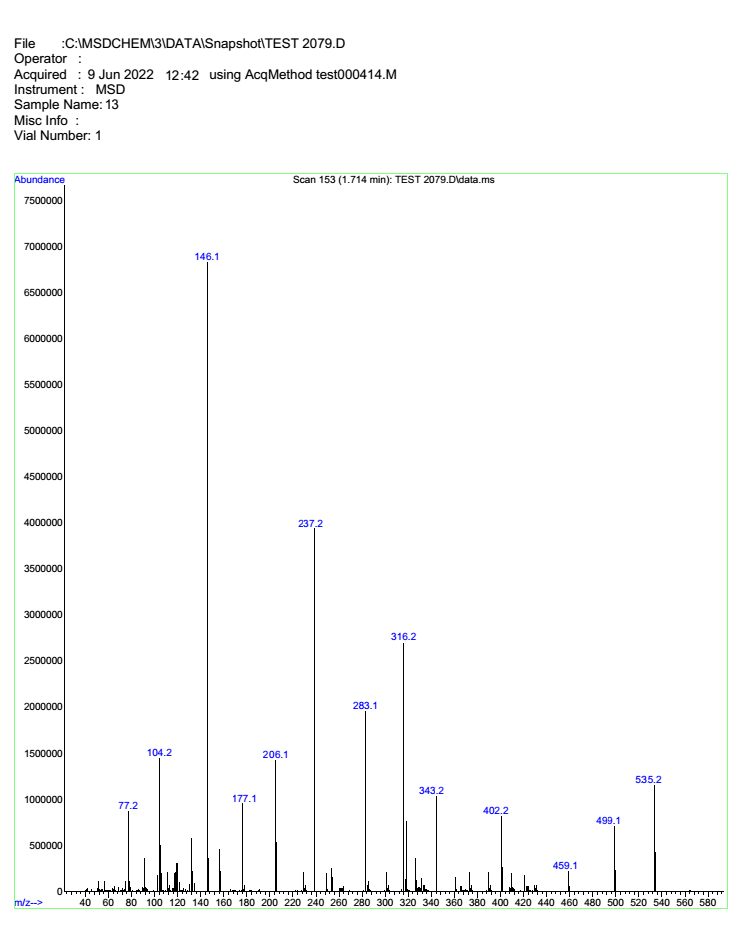


**Fig. S16.** Mass of 3-benzyl-2-(4-((1-(4-chlorobenzyl)-1*H*-1,2,3-triazol-4-yl)methoxy)phenyl)-2,3-dihydroquinazolin-4(1*H*)-one (10c)


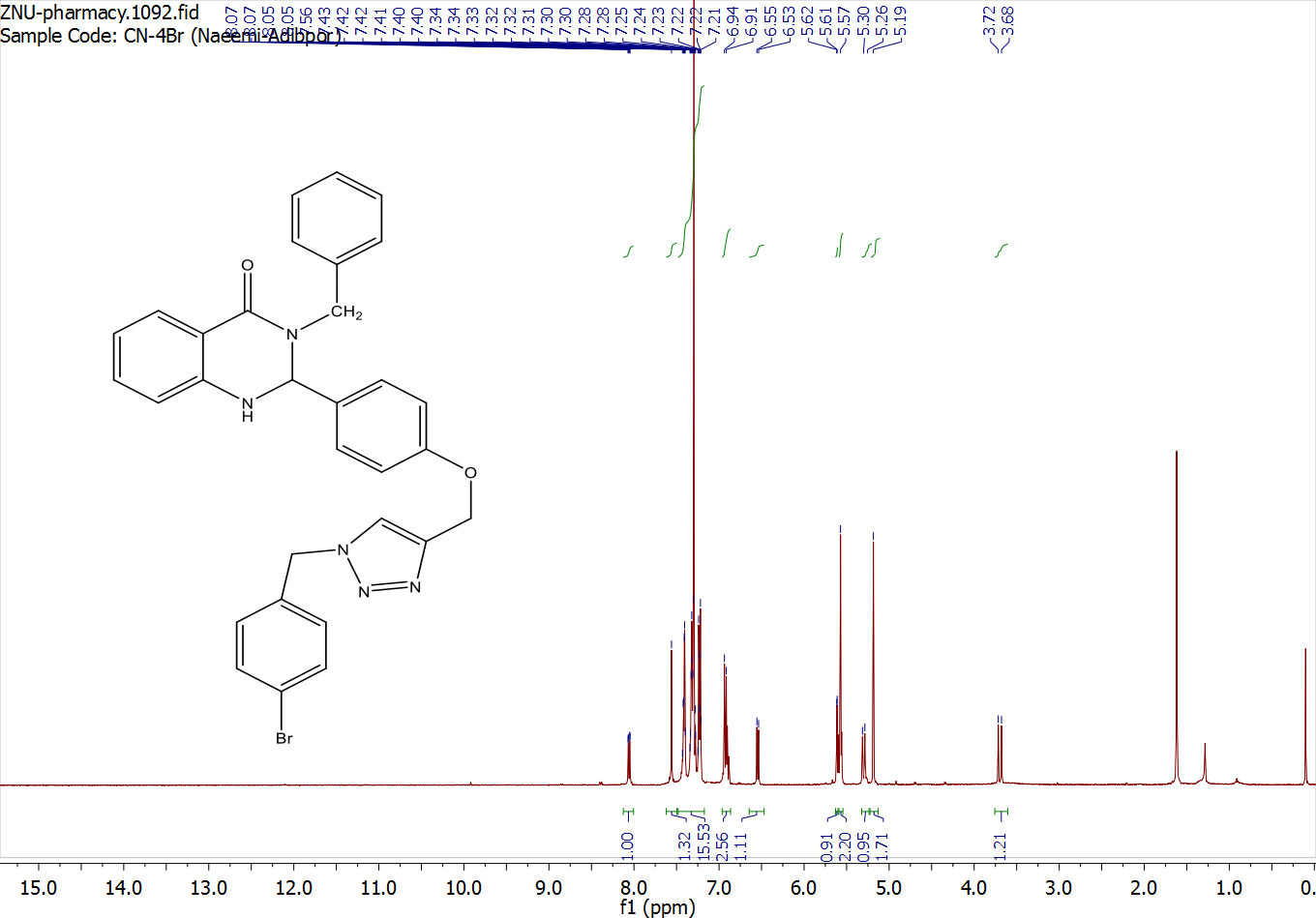


**Fig. S17.** ^1^H-NMR of 3-benzyl-2-(4-((1-(4-bromobenzyl)-1*H*-1,2,3-triazol-4-yl)methoxy)phenyl)-2,3-dihydroquinazolin-4(1*H*)-one (10d)


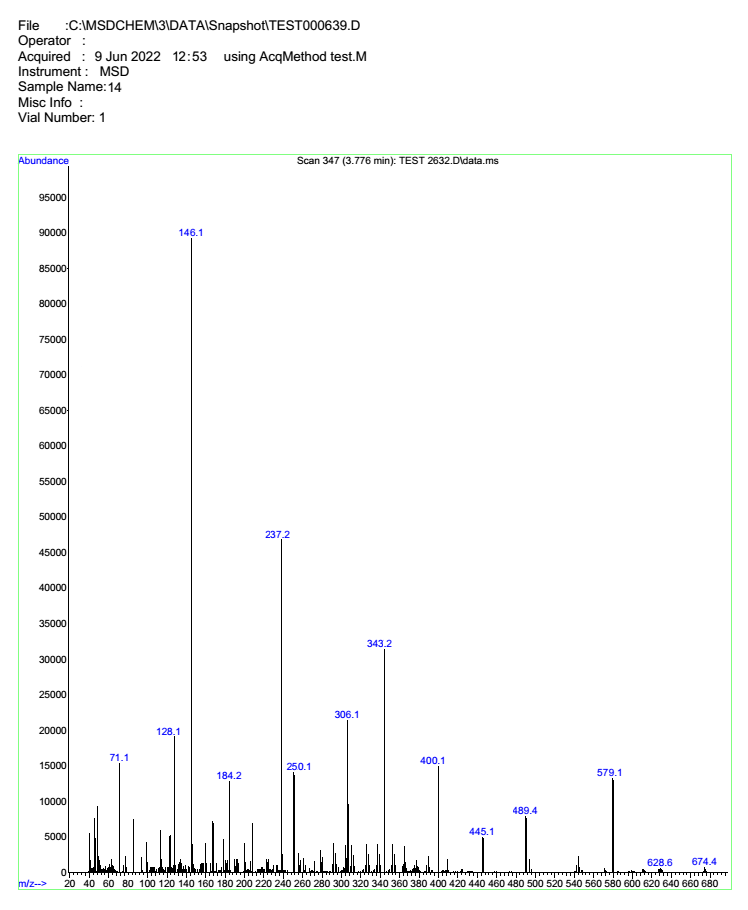


**Fig. S18.** Mass of 3-benzyl-2-(4-((1-(4-bromobenzyl)-1*H*-1,2,3-triazol-4-yl)methoxy)phenyl)-2,3-dihydroquinazolin-4(1*H*)-one (10d)


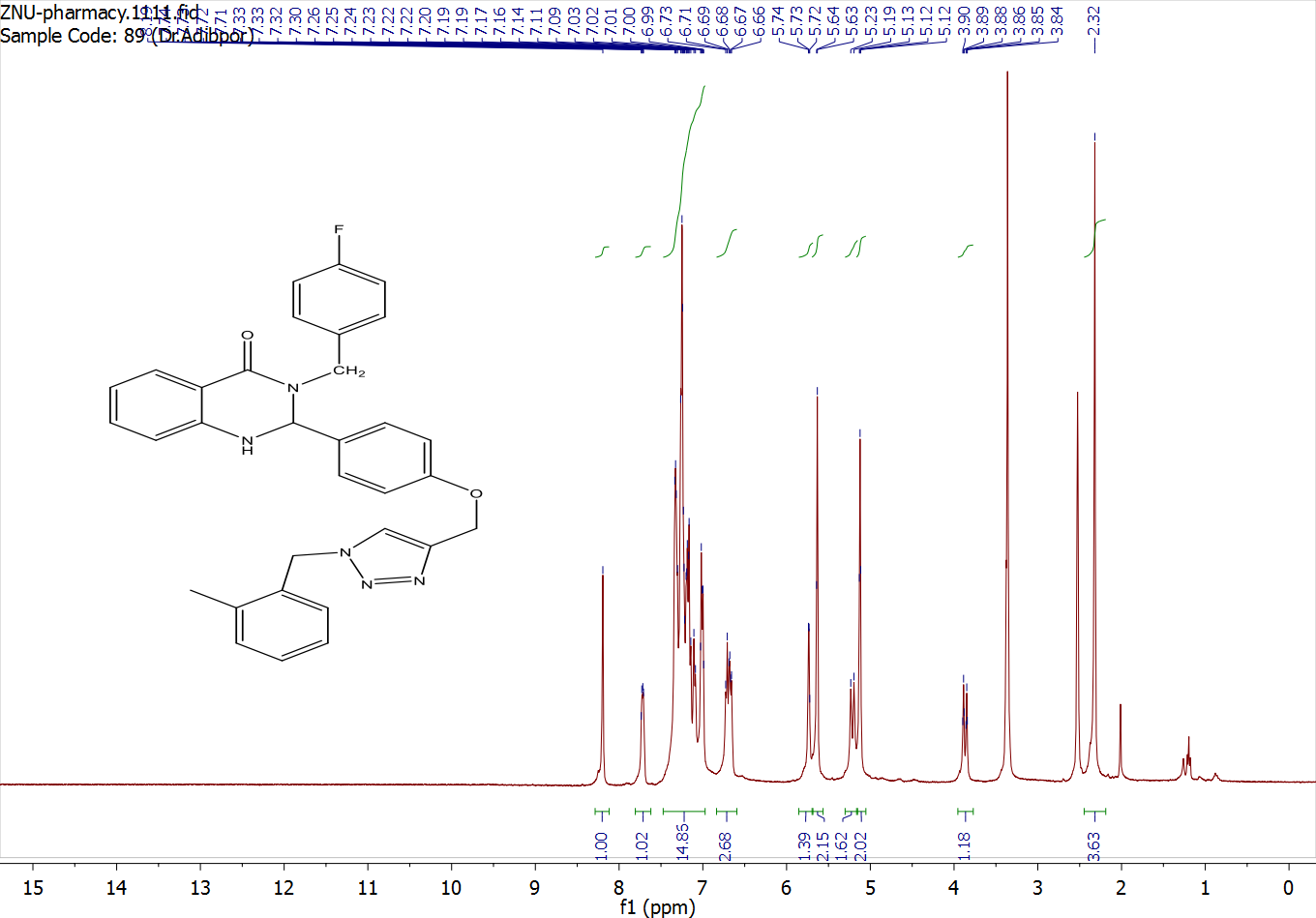


**Fig. S19.** ^1^H-NMR of 3-(4-fluorobenzyl)-2-(4-((1-(2-methylbenzyl)-1*H*-1,2,3-triazol-4-yl)methoxy)phenyl)-2,3-dihydroquinazolin-4(1*H*)-one (10e)


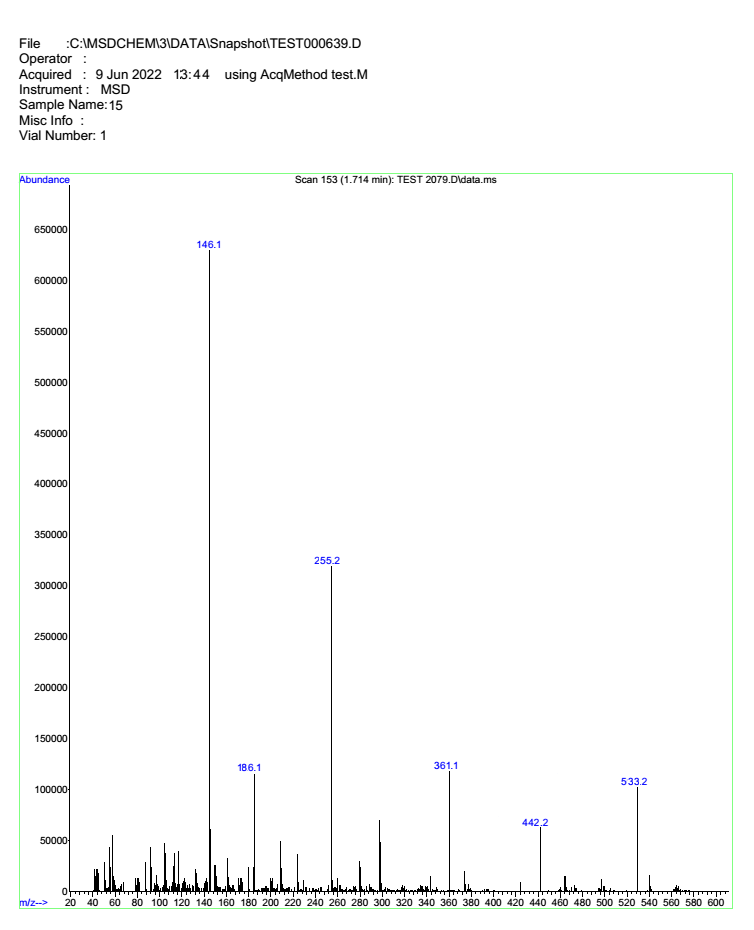


**Fig. S20.** Mass of 3-(4-fluorobenzyl)-2-(4-((1-(2-methylbenzyl)-1*H*-1,2,3-triazol-4-yl)methoxy)phenyl)-2,3-dihydroquinazolin-4(1*H*)-one (10e)


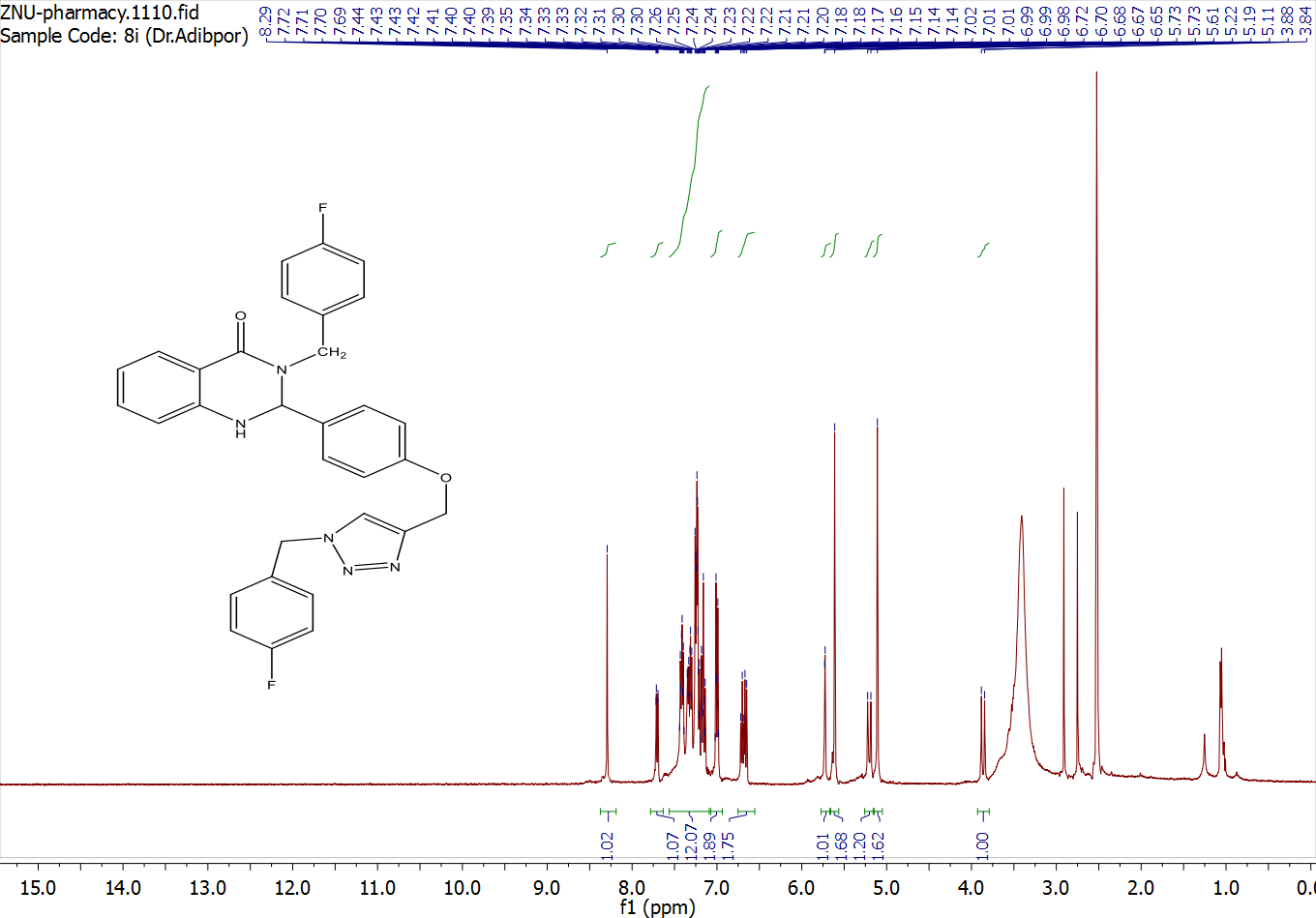


**Fig. S21.** ^1^H-NMR of 3-(4-fluorobenzyl)-2-(4-((1-(4-fluorobenzyl)-1*H*-1,2,3-triazol-4-yl)methoxy)phenyl)-2,3-dihydroquinazolin-4(1*H*)-one (10f)


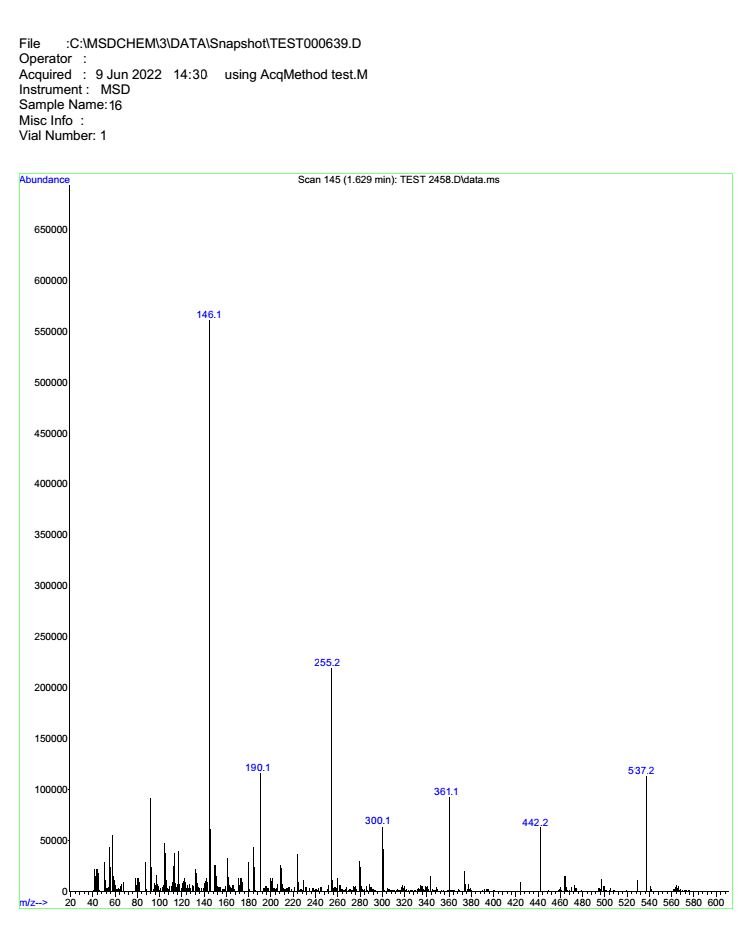


**Fig. S22.** Mass of 3-(4-fluorobenzyl)-2-(4-((1-(4-fluorobenzyl)-1*H*-1,2,3-triazol-4-yl)methoxy)phenyl)-2,3-dihydroquinazolin-4(1*H*)-one (10f)
